# Small molecule activator of Nm23/NDPK as an inhibitor of metastasis

**DOI:** 10.1038/s41598-018-29101-6

**Published:** 2018-07-19

**Authors:** Jae-Jin Lee, Hwang Suk Kim, Ji-Sun Lee, Jimin Park, Sang Chul Shin, Soonwha Song, Eunsun Lee, Jung-Eun Choi, Ji-Wan Suh, Hongsoo Lee, Eunice EunKyeong Kim, Eun Kyoung Seo, Dong Hae Shin, Ho-Young Lee, Hee-Yoon Lee, Kong-Joo Lee

**Affiliations:** 1 Graduate School of Pharmaceutical Sciences, College of Pharmacy, Ewha Womans University, Seoul, 03760 Korea; 2 Department of Chemistry, Korea Advanced Institute of Science & Technology, Daejeon, 34141 Korea; 3 College of Pharmacy and Research Institute of Pharmaceutical Sciences, Seoul National University, Seoul, 08826 Korea; 4 Biomedical Research Institute, Korea Institute of Science & Technology, Seoul, 02792 Korea

## Abstract

Nm23-H1/NDPK-A is a tumor metastasis suppressor having NDP kinase (NDPK) activity. Nm23-H1 is positively associated with prolonged disease-free survival and good prognosis of cancer patients. Approaches to increasing the cellular levels of Nm23-H1 therefore have significance in the therapy of metastatic cancers. We found a small molecule, (±)-trans-3-(3,4-dimethoxyphenyl)-4-[(E)-3,4-dimethoxystyryl]cyclohex-1-ene, that activates Nm23, hereafter called NMac1. NMac1 directly binds to Nm23-H1 and increases its NDPK activity. Employing various NMac1 derivatives and hydrogen/deuterium mass spectrometry (HDX-MS), we identified the pharmacophore and mode of action of NMac1. We found that NMac1 binds to the C-terminal of Nm23-H1 and induces the NDPK activation through its allosteric conformational changes. NMac1-treated MDA-MB-231 breast cancer cells showed dramatic changes in morphology and actin-cytoskeletal organization following inhibition of Rac1 activation. NMac1 also suppressed invasion and migration *in vitro*, and metastasis *in vivo*, in a breast cancer mouse model. NMac1 as an activator of NDPK has potential as an anti-metastatic agent.

## Introduction

The *Nm23* gene encodes nucleoside diphosphate kinase (NDPK), which catalyzes the exchange of terminal phosphate between different nucleoside diphosphates (NDP) and triphosphates (NTP) in a reversible manner to produce nucleoside triphosphates^1^. NDPK is a housekeeping enzyme for maintaining the equilibrium of different nucleoside triphosphates inside the cell^2^.

Nm23 consists of ten NME protein family members (Nm23-H1 to Nm23-H10). Nm23-H1 was first identified as a tumor metastasis suppressor protein^3^. Among the Nm23 family of genes, only Nm23-H1 and Nm23-H2, the most abundant Nm23 isoforms in the human cells, are known to suppress metastasis in multiple tumor types^4^. Loss of Nm23-H1 expression was shown to correlate with the degree of metastasis and prognosis in breast, ovarian, melanoma, gastric, and lung carcinoma^5–9^. Nm23-H1 suppresses metastasis by inhibiting multiple steps in the metastatic processes such as extravasation in primary site, extravasation, and metastasis colonization, and angiogenesis at secondary site^10^. Molecular mechanism of metastasis suppression of Nm23-H1 was elucidated by studies of the cytoskeleton-organizing pathway^11,12^, and among others, inhibiting MAPK signaling pathway through physical interaction with kinase suppressors of Ras (KSR-1/2)^13^. Nm23-H1 is known to possess multiple enzymatic activities including NDPK, protein histidine kinase, and 3′–5′ exonuclease^14,15^. Among these multiple enzyme activities, NDPK activity is crucial for Nm23-H1 mediated biological functions including cytoskeleton organization, insulin secretion, and endocytosis^11,13,16^. It has been suggested that Nm23-H2 suppresses tumor metastasis by influencing the expression of cell adhesion molecules such as vinculin and plakoglobin and their organization. Nm23-H2 expression is thus associated with the overall survival of lung cancer patients^17,18^.

There remains some controversy regarding the physiological relevance of NDPK activity of Nm23-H1 on tumor metastasis suppression^10^. However, most reports have demonstrated that NDPK activity of Nm23-H1 promotes metastasis suppression by supplying NTPs to cytoskeleton-organizing proteins such as EMMPRIN, dynamin and plackoglobin^11,12,18^. We also demonstrated that NDPK activity is crucial for tumor metastasis suppression of Nm23-H1 and that this activity is regulated by oxido-reduction system. Hexameric Nm23-H1 is required for suppression of tumor metastasis and it dissociates into dimers under oxidative conditions. The NDPK activity of hexameric Nm23-H1 is readily lost in response to mild oxidative stress which causes disulfide crosslinking^19^. The intra-disulfide crosslinking is reversibly regulated by oxido-reduction system via NADPH-thioredoxin reductase-thioredoxin^20^. The crystal structure of oxidized Nm23-H1 facilitatess the formation of an intramolecular disulfide bond between Cys4 and Cys145 that triggers a large conformational change and a suitable environment for the oxidation of Cys109 to sulfonic acid by stepwise oxidation and destabilizes the hexameric state^21^. The Nm23-H1 mutant C109A is not inhibited by oxidative stress, but shows constitutively active NDPK activity and suppresses invasion and migration in MDA-MB-231 breast cancer cells^20^. These studies confirmed that activation of NDPK leads to the inhibition or prevention of tumor metastasis.

Several approaches were used to increase the cellular level of Nm23-H1 to augment the suppression of tumor metastasis. Firstly, invasive ovarian cancer cells were infected with Nm23-H1 gene using an adenovirus-associated vector, to promote anti-metastasis and the prolongation of survival time^22^. Secondly, cell permeable Nm23 transduction has been proposed as adjuvant therapy against disseminated cancers^23^. Thirdly, since Nm23 expression is regulated by glucocorticoids, medroxyprogesterone acetate (MPA), an agonist of the glucocorticoid receptor, has been employed to increase the transcription of Nm23-H1 both *in vivo* and *in vitro*^24,25^. A phase 2 clinical study has been performed with MPA in metastatic hormone receptor negative breast cancer patients. However, no clear dose-response relationship was observed^26^. These unsuccessful attempts to increase cellular level of Nm23-H1 indicate that new approaches are needed to confirm the potential of increasing the cellular level of Nm23-H1 in preventing or arresting tumor metastasis.

In our efforts to meet this need, we looked for chemical approaches to enhancing the cellular activity of Nm23-H1 based on redox regulation of NDPK activity^20^. We screened a variety of natural product compounds having different scaffolds, activating NDPK activity (NMac) and found a small chemical NMac1 that acts as an NDPK activator. We further confirmed that NMac1 can inhibit tumor metastasis. We also identified the pharmacophore of NMac1, its mode of action and cellular function in inhibiting metastasis *in vivo* and *in vitro*.

## Results

### Screening for an NDPK activator

**Figure 1:**
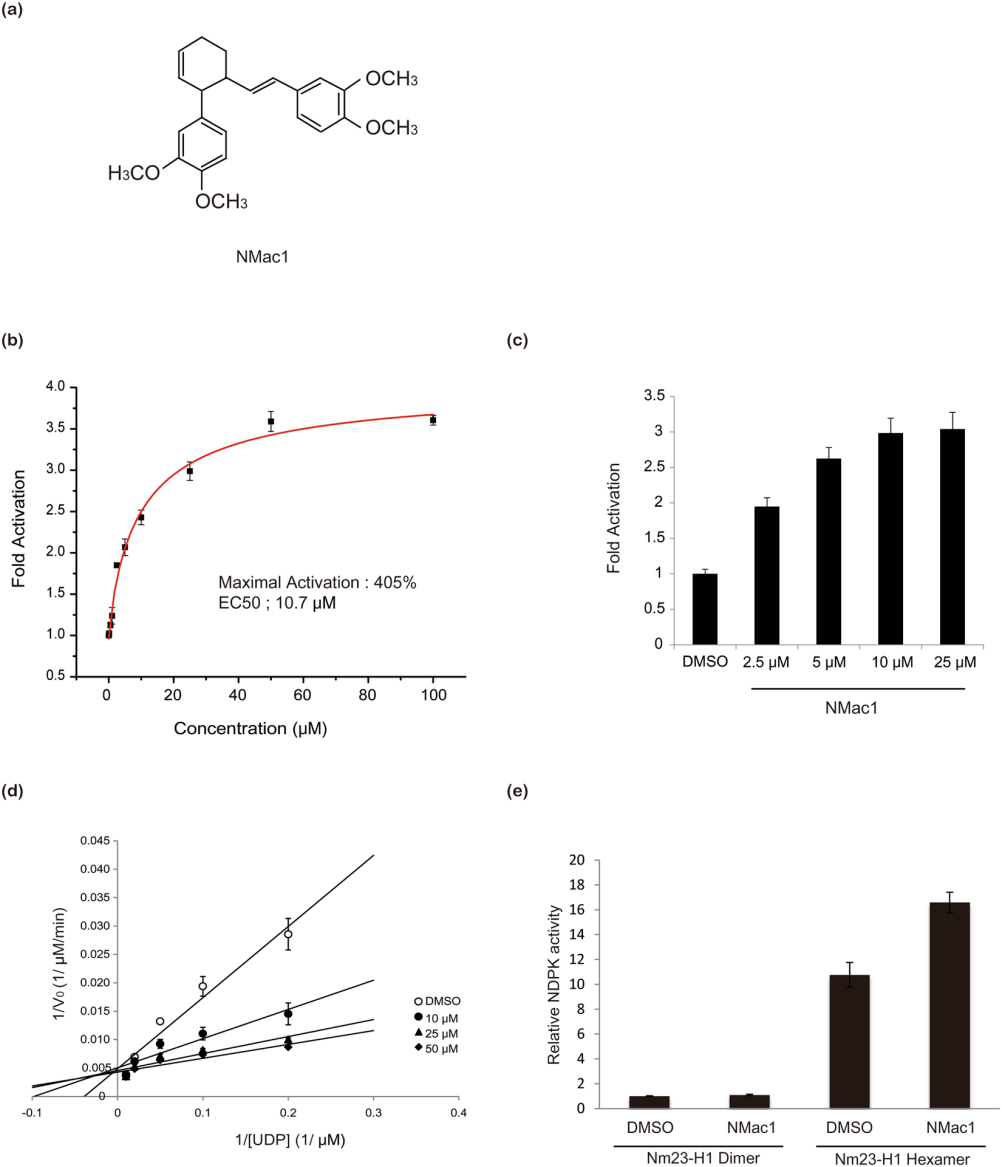
Identification and characterization of a small molecule NDPK activator of Nm23-H1 and H2. (**a**) Chemical structure of the NDPK activator, NMac1 (±)-trans-3-(3,4-dimethoxyphenyl)-4-[(E)-3,4-dimethoxystyryl]cyclohex-1-ene. (**b**) Effect of increasing concentrations of NMac1 on NDPK activity of recombinant human Nm23-H1. NDPK activity was measured by the amount of ATP produced by 5 ng of Nm23-H1 using 5 μM ADP and 5 μM UTP for 1 min with indicated concentration of NMac1, or 1% DMSO as vehicle. All experiments were triplicated, and data are expressed as mean ± S.D. (**c**) Cell based NDPK assay in a dose dependence. NDPK activity was measured by ATP consumption with 100 μg of MDA-MB-231 cell lysate, and 50 μM UDP. All experiments were triplicated, and data are expressed as mean ± S.D. (**d**) A double reciprocal plot with fixed concentration of NMac1 and various concentrations of UDP with 5 μM ATP. All experiments were triplicated, and data are expressed as mean ± S.D. (**e**) NDPK activation by NMac1 to Nm23-H1 hexamer and dimer which were fractionated using size exclusion chromatography. NDPK activity was measured by the amount of ATP produced by 5 ng of Nm23-H1 fraction using 5 μM ADP and 5 μM UTP for 1 min. All experiments were triplicated, and data are expressed as mean ± S.D.

Since Nm23-H1 has been identified as a tumor metastasis suppressor, increasing its levels to enhance the suppression of tumor metastasis has become a rational research goal. In order to meet this goal of finding potential activators of Nm23-H1 protein, we screened a variety of natural compounds having different scaffold that activate NDPK using recombinant Nm23-H1. These studies, in which NDPK activity was measured using radio-labeled UTP generation from radio-labeled ATP and non-labeled UDP as substrates, we identified a natural compound that activated NDPK activity of Nm23-H1 (Supplementary Fig. 1). This compound, isolated from *Zingiber cassumunar* Roxb. (Zingiberaceae)^27^, was identified as (±)-trans-3-(3,4-dimethoxyphenyl)-4-[(E)-3,4-dimethoxystyryl]cyclohex-1-ene, and hereafter called NMac1 (for Nm23 activator 1) (Fig. 1a). To measure the enzyme kinetics of Nm23-H1, we employed an optimized NDPK assay using ATP-dependent reaction catalyzed by firefly luciferase to measure ATP production by NDPK reaction and measured the NDPK activation of NMac1 with recombinant Nm23-H1, and cellular Nm23s. NMac1 potency was adjusted by determining the amount of the compound required for increasing enzyme activity by 50% (EC_50_) as well as the percentage maximum activation achieved. We found that NMac1 activated NDPK activity of recombinant Nm23-H1 in a dose dependent manner (Fig. 1b), EC_50_ was 10.7 µM and percentage maximum activation was 405% assessed by curve fitting. NMac1 also activated cellular NDPKs in MDA-MB-231 cells (Fig. 1c). These results confirm that NMac1 is a potent activator of NDPK proteins.

Since NMac1 is a racemic mixture, we examined which stereoisomer is more potent employing 3R-(3,4-dimethoxyphenyl)-4S-[(E)-3,4-dimethoxystyryl]cyclohex-1-ene (NMac2) and 3S-(3,4-dimethoxyphenyl)-4R-[(E)-3,4-dimethoxystyryl]cyclohex-1-ene (NMac3) enantiomers. The chirality of the molecule did not affect the activation of Nm23-H1 as shown in Supplementary Fig. 2A. In order to characterize the activation mechanism of human NDPK enzyme by NMac1, we examined the effect of NMac1 on the Vmax and the Km of NDPK for its substrate, UDP at fixed ATP concentration. NMac1 decreased the Km value of Nm23-H1 for substrate UDP without affecting Vmax (Fig. 1d). This activation could be achieved by increasing NDP substrate affinity via allosteric mechanism. A similar Km-type activation mechanism has been reported for small molecule activators of glucokinase and SIRT1^28,29^. To determine whether NMac1 activates dimeric or hexameric form of Nm23-H1, we examined the NDPK activation of identical amounts of each form purified by size exclusion chromatography, in response to NMac1. As shown in Fig. 1e, NMac1 activates NDPK activity of only hexameric Nm23-H1, but not dimeric one. We established the specificity of the effects of NMac1 on Nm23-H1 and H2, by showing that they were similar to the activation of purified recombinant Nm23-H1 and -H2 by NMac1 (25 μM) (Supplementary Fig. 2B). These results suggest that NMac1 activates the NDPK activity of hexameric form of both Nm23-H1 and –H2 via increasing the substrate affinity with allosteric mechanism.

### Structure activity relationship (SAR) of NMac1 for activating NDPK

**Figure 2:**
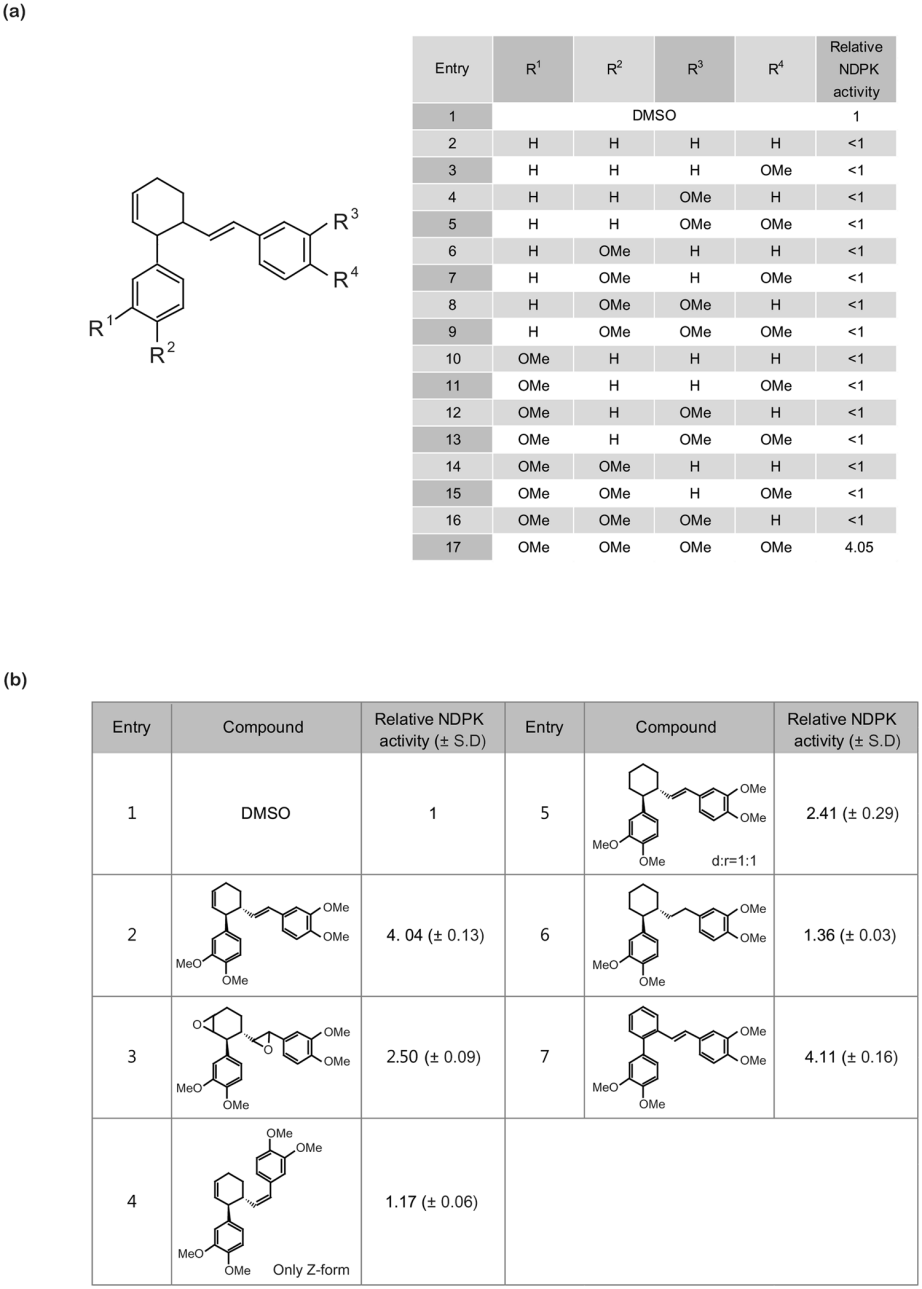
Structure activity relationship (SAR) of NMac1 derivatives for activating NDPK (**a**) Structure Activity Relationship (SAR) of catechol derivatives. (**b**) SAR around cyclohexene ring related.

To elucidate the pharmacophore of NMac1 for activating Nm23-H1, we designed and synthesized a number of NMac1 derivatives and assessed their abilities to activate NDPK activity (Fig. 2a). First, we investigated the effect of methoxy groups on the both catechol rings of NMac1. We altered the number of methoxy groups in the structure of NMac1. Deletion of methoxy group in any position on catechol ring inhibited NDPK activity of Nm23-H1. Only NMac1 which contains all four methoxy groups showed activation of the Nm23-H1. While each methoxy group of NMac1 may affect the binding affinity to Nm23-H1, all four methoxy groups are essential for activation of Nm23-H1. Next, we studied the effect of the relative positions of the two catechol rings connected by cyclohexene ring and olefin chain. We investigated whether those structural motifs regulate the geometrical conformation of compound (Fig. 2b). When the unsaturated C-C bonds on cyclohexene ring and olefin were altered slightly to epoxides (entry 3), this decreased the NDPK activity slightly. The (Z)-isomer of NMac1 abolished the increase of NDPK activity (entry 4). When the cyclohexene ring was reduced to cyclohexane ring, the derivative showed decreased activity (entry 5). These results along with the similar activity of NMac1 enantiomers indicated that the two catechol rings need preferably to be located near the same plane. Also, the derivative with the fully saturated side chain did not exhibit the increased activity (entry 6). Since reduction of cyclohexene ring into the cyclohexane ring altered the location of the two catechol rings from near the same plane into the skewed 3D-positoin, the 3D-location of two catechol rings in NMac1 are essential for the activation of NDPK and the two rings should be near the same plane in the space for the optimal activity. This pharmacophoric requirement for NMac1 is strongly supported by high activities shown by derivatives of NMac1 with phenyl replacement of the cyclohexene ring (entry 7).

### NMac1 directly binds to the C-terminal region of Nm23-H1

In order to investigate the mode of action of NMac1, we first examined NMac1 binding to Nm23-H1, employing surface plasmon resonance (SPR) analysis. The bindings of NMac1 to recombinant Nm23-H1 were measured with and without ATP with SPR. Relative responses of binding were increased in a dose-dependent manner (Supplementary Fig. 3). The *K*_*D*_ values of NMac1 to Nm23-H1 in the presence and absence of ATP, were determined by SPR analysis as shown in Table 1. The dissociation constant Kd of NMac1 to Nm23-H1 in the presence of ATP was ten-fold lower than that in the absence of ATP, while the association constant Ka’s are similar regardless of the presence or absence of ATP. These results indicate that NMac1 directly binds to Nm23-H1 and ATP binding in active site reduces the dissociation of NMac1 from Nm23-H1.

**Table 1:** Binding kinetics between Nm23-H1 and NMac1.

Ligand	K_a_^a^ [M^−1^ s^−1^]	Kd^a^ [s^−1^]	K_D_^b^ [M]
NMac1	78 ± 1	1.39 ± 0.01 e^−3^	17.9 ± 0.2 μM
NMac1, 2 mM ATP	71 ± 1	1.06 ± 0.07 e^−4^	1.49 ± 0.03 μM

^a^Obtained by saturated binding responses averaging at least three independent runs of SPR measurements.

^b^K_D_ = K_d_/K_a._

**Figure 3:**
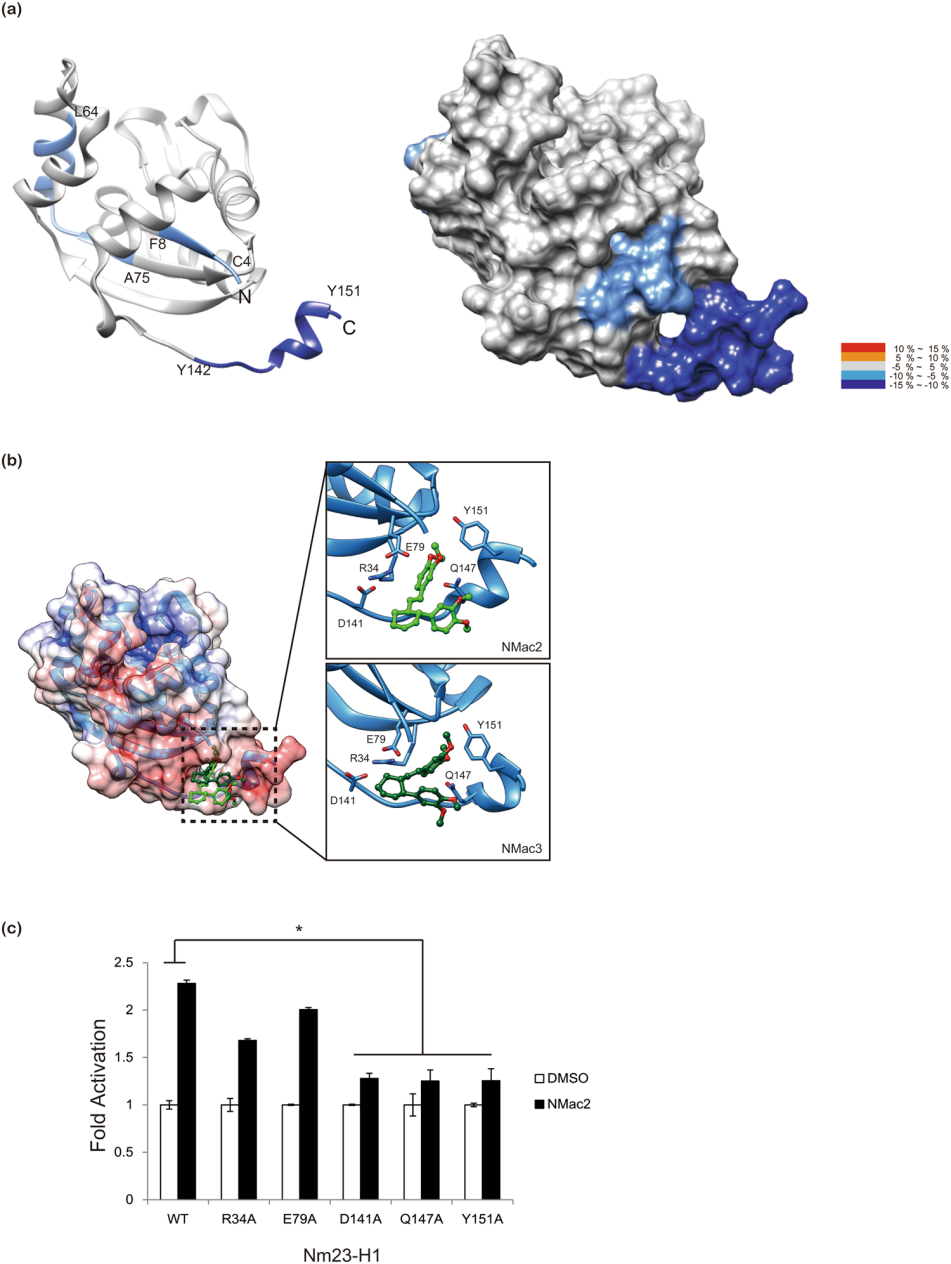
NMac1 binds to C-terminal of Nm23-H1. (**a**) Overlay of differential HDX data of NMac1 bound to Nm23-H1. This overlay compares HDX of *apo* Nm23-H1 with that of NMac1 bound Nm23-H1. Perturbation data are color coded and plotted onto the backbone of the 1JXV PDB file according to the key. Observed changes in HDX were statistically significant (p < 0.05) in a two tailed t-test (n = 3) (**b**) Molecular modeling of interaction between Nm23-H1 and chiral isomer NMac2 and NMac3 (**c**) Nm23-H1 C-terminal define the allosteric compound binding site. The activity of NMac2 to activate NDPK was examined against a series of Nm23-H1 mutants.

Next, in order to identify the binding region of NMac1 with Nm23-H1 and its mode of action, we employed hydrogen/deuterium exchange-mass spectrometry (HDX-MS), which measures the differential deuterium incorporation in proteins and peptides with or without ligand and is a powerful tool to elucidate ligand binding and ligand-mediated structural changes^30^. Differential deuterium incorporations of identified peptides of Nm23-H1 by NMac1 binding were listed in a time dependent manner in Supplementary Table 1. Discernible changes were occurred in three peptic peptides; a.a 2–8, 64–75, and 142–152 residues and presented in the structure of Nm23-H1 monomer (Fig. 3a). HDX in peptides (a.a 2–8 and 142–152) were significantly decreased by NMac1 binding and these peptides were turned out as small pocket in C-terminal region of Nm23-H1, indicating that NMac1 interacts with C-term of Nm23-H1 as shown in Fig. 3a. Also these peptides are involved in intra-disulfide linkage (Cys4-Cys145) formation by oxidation^21^. The hexameric structure presented in Supplementary Fig. 4 shows that peptide (a.a 64–75 residue) is located in the boundary between C-terminal region and adjacent monomer. These results suggest that NMac1 binds to C-terminal of Nm23-H1 altering adjacent residues inter (a.a 2–8)/intra (a.a 64–75) peptides of Nm23-H1 hexamer.

Next, we carried out *in silico* docking studies to understand the structural basis of NMac1 interactions in the C-terminal of Nm23-H1. The *in silico* docking model indicated that its chiral activators (NMac2 and NMac3) that have similar NDPK activation shown as Supplementary Fig. 2A are located at the small pocket composed of the N- and C-terminal loops. The binding modes of NMac2 and NMac3 are represented in Fig. 3b, and Supplementary Fig. 5. Among the key interactions between NMacs and Nm23-H1, methoxy group in ring A directly connected to cyclohexene ring was predicted to form hydrogen bond with Q147. In order to verify this prediction, NDPK activation of Nm23-H1 by NMac2 was investigated employing Nm23-H1 mutants at the predicted binding region (R36A, E39A, D141A, Q147A, Y151A). As shown in Fig. 3c, NMac2 activated Nm23-H1 mutants R36A and E39A as well as WT at expected levels, while activating in mutants D141A, Q147A, and Y151A at significantly decreased levels. Thus, these mutant studies confirm the predicted binding modes at C-terminal. SPR studies showed that ATP stabilized NMac1 binding to Nm23-H1, and we observed minor differential deuterium exchange in Kpn-loop (a.a 96–116) by NMac1 binding (Supplementary Table 1). These results are consistent with previous reports that C-terminal region of Nm23-H1 plays key roles in structural stability and NDPK enzymatic activity by participating in the interaction between subunit having Kpn-loop and nucleotide binding site in *Leishmania parasites*^31^. NMac1 slightly affects hexamer formation of Nm23-H1 (Supplementary Fig. 6).

### NMac1 induces morphological changes in highly invasive breast cancer cells via actin-cytoskeleton reorganization

**Figure 4:**
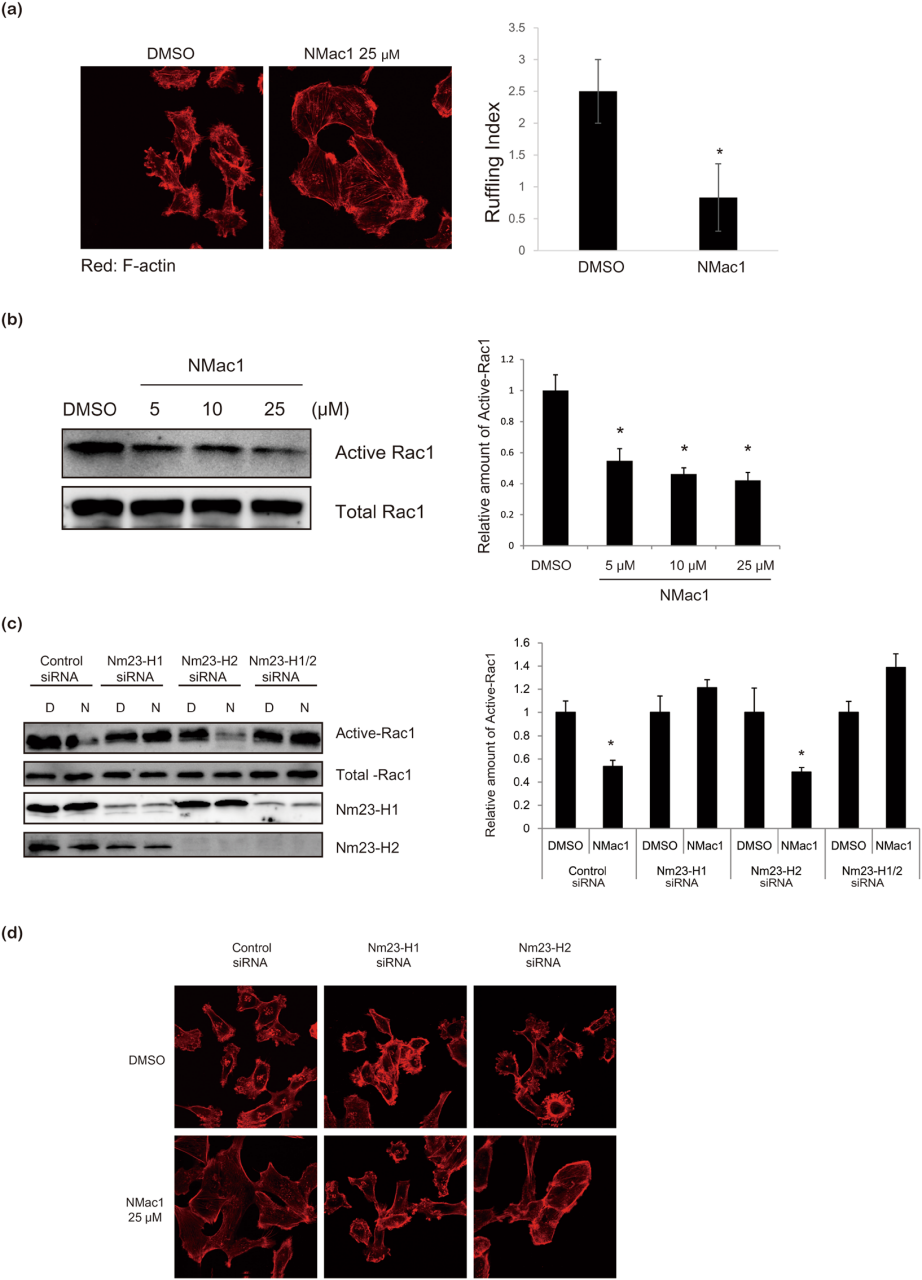
NMac1 induces morphological and other changes in MDA-MB-231 cells. (**a**) NMac1 reduces membrane ruffle in MDA-MB-231 cells. Localization of F-actin was analyzed by confocal microscopy in control and NMac1 treated cells. MDA-MB-231 cells treated with NMac1 25 µM (or 0.05% DMSO as vehicle) for 16 h were stained by phalloidin-Rhodamine. Number of cell ruffles of MDA-MB-231 cells was quantified by determining the ruffling index. (**b**) NMac1 reduces Rac1 activation. Active Rac1 pulldown assay was conducted in control and NMac1 treated MDA-MB-231 cells with indicated concentrations for 16 h. A representative experiment out of 3 independent experiments is shown. (**c**) NMac1 reduces Rac1 activation via Nm23-H1. Active Rac1 pulldown assay was conducted in control and NMac1 treated Nm23-H1 or (/and) H2 knocked down MDA-MB-231 cells with indicated concentrations for 16 h. A representative experiment out of 3 independent experiments is shown. (**d**) NMac1 reduces membrane ruffle in MDA-MB-231 cell via Nm23-H1. Localization of F-actin was analyzed by confocal microscopy in control and NMac1 treated Nm23-H1 or/and H2 knocking down MDA-MB-231 cells. At 48 hours after Nm23-H1 or/and H2 knocking down, MDA-MB-231 cells were treated with NMac1 25 µM (or 0.05% DMSO) for 16 hours followed by staining with phalloidin-Rhodamine.

In order to investigate cellular effects of NMac1 treatment, we examined the morphological changes following treatment with NMac1, in a highly invasive breast cancer cell line, MDA-MB-231 cells which express low levels of Nm23-H1. The morphological changes observed in MDA-MB-231 cells following NMac1 treatment included substantial alteration of the original highly mesenchymal type having a lot of ruffles to a non-metastatic morphology having reduced ruffles and increased cell-covered area (Fig. 4a). This is in agreement with previous reports that overexpression of Nm23-H1 promotes adhesion of cancer cells^32^, and silencing of Nm23-H1 induces the invasive phenotype showing increased cellular motility, directional migration, and reorganization of actin cytoskeleton regulated by small G-protein Rac1^33^. Because Nm23-H1 is known to inhibit Rac1 GTPase activation by association with guanidine nucleotide exchanging factor (GEF) for Rac1, Tiam1^34,35^, we examined Rac1 activation in MDA-MB-231 cells after treatment with various concentrations of NMac1. As shown in Fig. 4b, NMac1 specifically inhibited active Rac1-GTP formation with an EC_50_; 10–15 μM under the assay conditions, without affecting the activities of other small G-protein RhoA (Supplementary Fig. 7A). We also examined the morphological changes of MDA-MB-231 cells in response to Rac1 inhibitor, NSC23766. As shown in Supplementary Fig. 7B, Rac1 inhibition with NSC23766, induced morphological changes similar to NMac1 treatment. This suggests that the morphological change by NMac1 is mediated by Rac1 inactivation. We excluded the possibility that the shape changes by NMac1 caused epithelial–mesenchymal transition (EMT), because EMT markers were not altered in response to NMac1 treatment (Supplementary Fig. 7C). Next, we investigated whether NMac1 inhibits the Rac1 activation specifically via Nm23-H1 or H2, by examining Rac1 activation by NMac1 in MDA-MB-231 cells after knocking down Nm23-H1 or (/and) H2. As shown Fig. 4c, NMac1 specifically inhibited Rac1 activation in control siRNA and Nm23-H2 siRNA cells, while Rac1 activation in Nm23-H1 silenced cells was not affected by NMac1 treatment. Moreover, NMac1-induced actin-cytoskeleton reorganization was abolished in cells in which only Nm23-H1 was knocked down (Fig. 4d). These results indicate that NMac1 inhibits Rac1 activation through NDPK activation of Nm23-H1.

### NMac1 suppressed breast cancer metastasis both in *in vitro* and *in vivo* models

**Figure 5:**
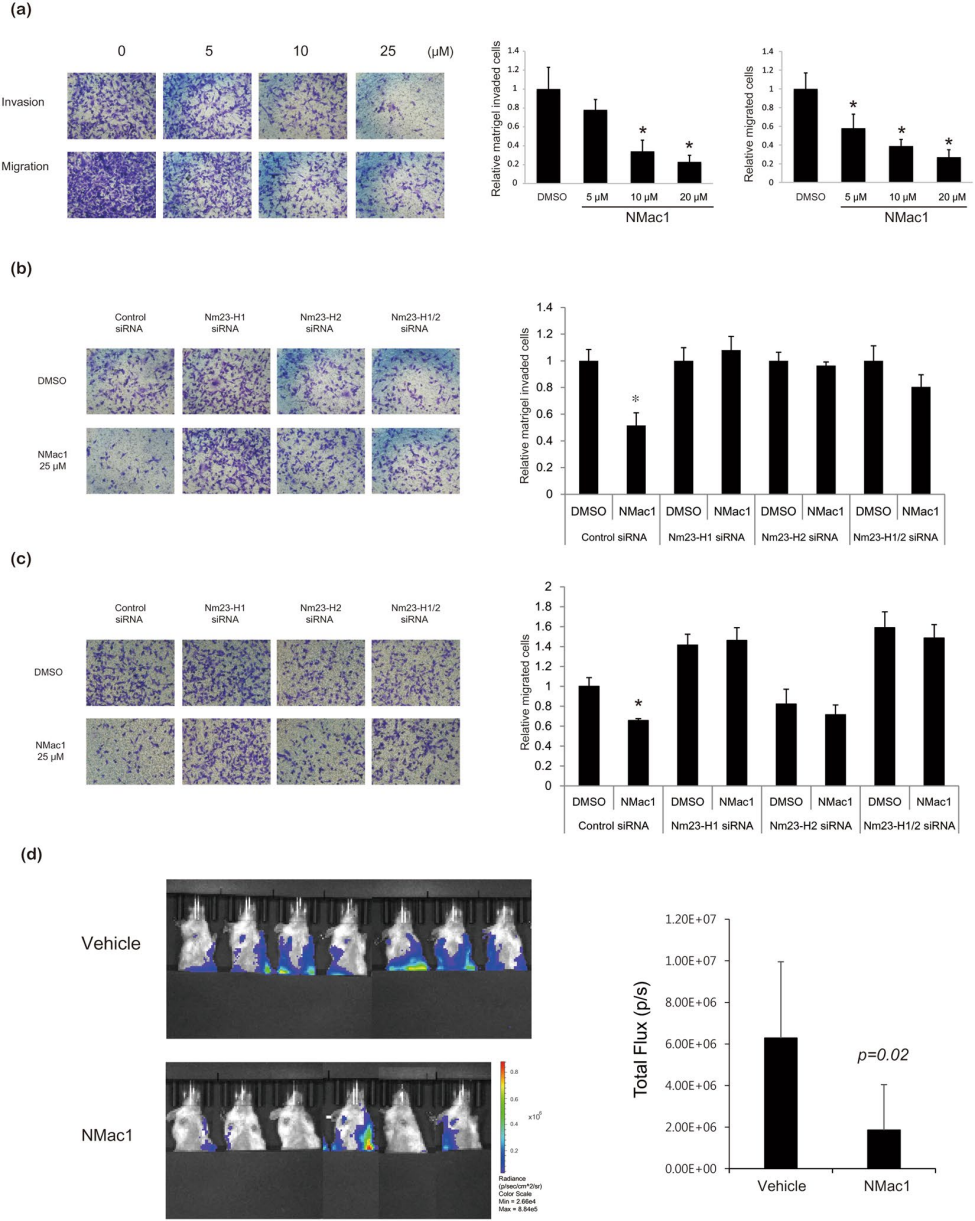
Effects of NMac1 on *in vitro* invasion/migration of MDA-MB-231 cells and *in vivo* metastasis. (**a**) NMac1 inhibits transwell migration and matrigel invasion of MDA-MB-231 cells. MDA-MB-231 cells treated with indicated concentrations of NMac1 were allowed to migrate in Boyden chambers for 24 hours. Bars indicate percentage of migration and invasion. ^*^P < 0.05, Student’s t-test. (**b**) NMac1 inhibits matrigel invasion of MDA-MB-231 cells via Nm23-H1 and H2. Nm23-H1 or (/and) H2 knocking down MDA-MB-231 cells treated with 25 µM of NMac1 were allowed to invaded in matrigel coated Boyden chambers for 24 hours. Bars indicate amount of migration and invasion. ^*^P < 0.05, Student’s t-test. (**c**) NMac1 inhibits transwell migration of MDA-MB-231 cells via Nm23-H1 and H2. Nm23-H1 or (/and) H2 knocking down MDA-MB-231 cells treated with indicated concentrations of NMac1 were allowed to migrated in Boyden chambers for 24 hours. Bars indicate percentage of migration and invasion. ^*^P < 0.05, Student’s t-test. (**d**) MDA-MB-231-Luc-D3H2LN cells were orthotopically injected into NOD/SCID mice and the mice were treated with vehicle or NMac1 (n = 7 for VEH group, n = 6 for NMac1 group). (Left) Bioluminescence images visualizing the metastatic tumor cells. (Right) Quantification of bioluminescence images 3 weeks after the NMac1 treatment. Data are presented as mean total photon flux per second ± S.D.

Because of the finding that NMac1 treatment induces Rac1 inhibition and reorganization of actin cytoskeleton, we examined the migration and matrigel invasion of *in vitro* breast cancer cell lines in response to NMac1. Highly metastatic breast cancer cell line MDA-MB-231 was treated with NMac1 and the transwell migration and matrigel invasion of the treated cells were assessed. As shown in Fig. 5a, NMac1 significantly reduced the invasion and migration of MDA-MB-231 cells in a dose dependent manner, without affecting their proliferation (Supplementary Fig. 8A). To investigate whether NMac1 inhibits the cell migration mediated by activating NDPK activities of Nm23-H1 or H2 in MDA-MB-231 cell line, we examined the cell invasion (Fig. 5b) and migration (Fig. 5c), and proliferation of MDA-MB-231 cells in which Nm23-H1 or (/and) H2 was silenced (Supplementary Fig. 8B). We found that NMac1 only inhibited invasion and migration of control siRNA transfected MDA-MB-231 cells, while the migration and invasion of Nm23-H1 knocked down MDA-MB-231 cells were not affected by NMac1 treatment. These results indicate that NDPK activation of Nm23-H1 by NMac1 treatments is crucial for suppression of invasion and migration of breast cancer cells. Intriguingly, *in vitro* invasion and migration of MDA-MB-231 cells were dramatically reduced by Nm23-H2 knocking down itself; however, NMac1 effects that reduce invasion and migration were not observed. This result is consistent with previous reports whether Nm23-H2 suppresses tumor metastasis is controversial, and NDPK activity of Nm23-H2 on tumor metastasis suppression remains to be elucidated.

The metastasis phenotype relies on the ability of tumor cells to adhere to distal site. The therapeutic potential of NMac1 to inhibit the metastasis of cancer was tested *in vivo* breast cancer metastasis mouse model. MDA-MB-231-Luc-D3H2LN cells, a luciferase expressing metastatic human breast cancer cell line, were injected (1 × 10^6^) into the mammary fat pad of 5-week-old, non-obese diabetic (NOD)/severe combined immune-deficient (SCID) mice, and after primary tumor size reached 100 mm^3^, the mice were treated daily with vehicle or 10 mg/Kg NMac1 for 3 weeks. Figure 5d shows that NMac1 significantly inhibited breast cancer metastasis *in vivo*, without affecting the primary tumor size (Supplementary Fig. 9A), and body weight (Supplementary Fig. 9B). This is in agreement with the previous report that Nm23-H1 significantly reduces metastasis without affecting primary tumor size^36^. These findings collectively confirm that NMac1 inhibits metastasis both *in vitro* and *in vivo* models and suggest that NMac1 has therapeutic potential as an anti-metastasis agent for metastatic breast cancer.

## Discussion

In the present study, we identified a small molecule NDPK activator, NMac1, by screening a variety of natural product compounds. We also examined the structure-activity relationships of various synthetic derivatives of NMac1 and elucidated the activator’s pharmacophore. We also investigated the mode of action of NMac1 on Nm23-H1 employing HDX-MS and mutation studies. We found that the NMac1 binding on C-terminal region of Nm23-H1 causes allosteric regulation of nucleotide binding and activates the NDPK activity and that NMac1 significantly reduces cell invasion *in vitro* via Rac1 inhibition and cancer metastasis *in vivo*. These studies suggest the potential of NMac1 as anti-metastatic agent for breast cancer.

Nm23-H1 was previously identified as a metastasis suppressor protein, regulating multiple stages in the metastasis process. Several attempts were made to increase Nm23-H1 and reduce the invasion of metastatic cancers, employing various biological approaches including MPA-induced Nm23-H1 overexpression, adenovirus-associated Nm23-H1 gene transfer, and cell permeable Nm23-H1 transduction^22–24^. These biological approaches were successful to varying degrees in increasing Nm23-H1 and suppressing the cancer metastasis. The present study is the first reported use of a small molecule to activate the Nm23-H1 function and prevent the breast cancer metastasis.

*Zingiber cassumunar* Roxb has been used as anti-inflammation agents in East Asia^37^, and isolated compounds from *Zingiber cassumunar* Roxb have been reported as having anti-inflammatory, and anti-metastatic activity^38,39^. NMac1 isolated from *Zingiber cassumunar* Roxb. (Zingiberaceae)^27^, and previously reported that inhibits p-glycoprotein^40^, and induces apoptosis to lung cancer cells^41^, however molecular targets of NMac1 were not addressed in these reports. Although, cumulated reports that similar structured natural compounds having anti-cancer and anti-inflammation activity, as we have shown in SAR, NMac1 have optimal structure to activate Nm23 proteins and its derivatives reduce its activity. Pharmacophore of NMac1 represents that all four methoxy groups of two dimethoxy phenyl rings are crucial for NDPK activation, and relative position of the two dimethoxy phenyl ring is also important. Based on the SAR, selective, potent and novel NDPK activators for Nm23-H1 remain to be further investigated.

NMac1 showed Km type activation that increases the affinity of NDP substrates by interacting with C-terminal of Nm23-H1. Enzyme activators indispensably take allosteric mechanism that activator binding region structurally regulates active site to increase enzyme activity which is distinct with competitive enzyme inhibitors. Glucokinase activator and SIRT1 activators (STACs) have been reported as Km type activator as well^28,29^, and STACs have been well elucidated as allosteric activator by HDX experiments and mutation study^42^. ATP binding stabilizes NMac1 interaction with Nm23-H1 in SPR studies. These results indicate the allosteric regulation between C-terminal loop and nucleotide interaction of Nm23-H1. A Kpn-loop of Nm23 is crucial for NDPK activity which makes up the nucleotide binding site. This study agrees with oxidized Nm23-H1 structure that C-terminal loop distortion induced by Cys4-Cys145 intramolecular disulfide reflects C109 oxidation followed by destabilization of hexamer^21^. Recent study also reveals that C-terminal region is cooperative with Kpn-loop to maintain hexamer of Nm23 protein^31^.

NMac1 induces remarkable morphological changes in mesenchymal breast cancer cell line, MDA-MB-231, conversion to epithelial shape having significantly reduced membrane ruffle, increased cell to cell contact, and cell adhesion. This phenotype is mainly mediated by Nm23-H1, and is consistent with recent reports that established the unique role of Nm23-H1 in adherence junction, cell morphology, and actin-cytoskeleton reorganization^11,33^. Collectively, this study provides the evidence of a role of NDPK activity and NMac1 effect on tumor metastasis especially actin cytoskeleton reorganization and cell adhesion.

In summary, this study reports the discovery of a small molecule NDPK activator, that we named NMac1, which acts as a metastasis suppressor and describes the pharmacophore and mode of action of NMac1 on Nm23-H1. Moreover, this study suggests the possibility of usage of NMac1 in combination therapy with anti-tumor agents in breast cancer.

## Methods

### Nm23-H1 Protein expression and purification

Nm23-H1 was expressed and purified as previously described^21^. Briefly, cytosolic fraction of *E. coli* strains BL21 (DE3) transformed with pET-3c Nm23-H1/H2 were obtained after inducing the expression of each protein with 1 mM isopropyl-D-thiogalactopyranoside (IPTG). Each cytosolic fraction was applied to ATP-sepharose column equilibrated with Buffer A (20 mM Tris-acetate, 20 mM NaCl, 0.1 mM EDTA, 3 mM MgCl_2_, pH 7.4). Then Nm23-H1 was eluted with Buffer A containing 1 mM ATP. Nm23-H1 mutants were generated by site-directed mutagenesis kit by supplier instruction (Promega).

### NDPK assay

*In vitro* NDPK assay; 5 ng of recombinant Nm23-H1 were pre-incubated with 5 µM GTP (or UTP) and NMac1 in NDPK assay buffer (20 mM HEPES, 3 mM MgCl_2_) at R.T. for 10 min followed by enzyme reaction by adding 5 µM for 1 min. Enzyme reaction was stopped by 95 °C boiling for 10 min. ATP generations were assessed by ATP determination kit (Molecular probe, USA). Cell based NDPK assay was performed. Cell lysates obtained from 5 × 10^6^ MDA-MB-231 cells which were lysed with NDPK assay buffer with protease inhibitor cocktail, and centrifuged 8,000 rpm at 4 °C for 10 min. 40 µL lysate incubated with NMac1 for 5 min followed by NDPK reaction by adding 50 µM UDP. ATP consumption was assessed by ATP determination kit (Molecular probe, USA). Origin 6.0 (USA) was used for curve fitting of NDPK assay data with logistic dose responsive function.

### Synthesis of NMac1 derivatives

NMac1 and its derivatives were prepared following the literature procedures (Supplementary Fig. 10a). Diels-Alder reaction of (E)-1,3-diene and methyl acrylate followed by reduction and epimerization gave the *trans*-di-substituted cyclohexene scaffold. And olefination with the corresponding phosphonate provided (E)-selective NMac1 analogues successfully. For scalable synthesis of NMac1, we modified known procedures and obtained NMac1 in high yield (See Supplementary Fig. 11 for detailed procedures). Using above methods, we achieved the preparation of sixteen NMac1 derivatives by combinations of the existence of methoxy groups on catechol ring. Further manipulation of NMac1 was furnished by dimethyldioxirane (DMDO) oxidation to obtain bis-epoxide in moderate yield (Supplementary Fig. 10B). Also hydrogenation of NMac1 provided the product that both olefins in cyclohexene ring and styrene were reduced. The (Z)-isomer of NMac1 was also prepared from **7** (Supplementary Fig. 10C). Stork-Zhao olefination of **7** gave (Z)-vinyl iodide selectively and Suzuki cross-coupling with boronic acid provided (Z)-isomer of NMac1 in high yield. Analogs with phenyl replacement of the cyclohexene ring were prepared from *o*-bromobenzaldehyde through Suzuki-cross coupling with catechol boronic acid followed by the Wadsworth-Emmons olefination and hydrogenation (Supplementary Fig. 10D).

### Surface Plasmon Resonance analysis

Interaction of NMac1 with Nm23-H1 was analyzed at 25 °C using a surface plasmon resonance instrument SR7500 DC (Reichert Technologies, NY). Nm23-H1 (1 mg/mL) in 10 mM S.A buffer pH 4.5 was immobilized using the standard amino coupling at 20 µL/min for 10 min on a carboxymethyl dextran hydrogel (CMDH) surface sensor chip (Reichert Technologies, NY) until saturation was achieved. Different concentrations of NMac1 (10–160 µM) in binding buffer (10 mM HEPES pH 7.4, 100 mM NaCl, 1 mM MgCl_2_, and with (or without 2 mM ATP)) were allowed to flow over the surface containing immobilized Nm23-H1 (approximately 8200 RU) at a rate of 30 μL/min. The sensor surface was regenerated after each association and dissociation cycle by injecting 2 M NaCl for 1 min. Sensorgrams were fit to a simple 1:1 Langmuir interaction model (A + B ⇌ AB) using data analysis program Scrubber 2.0 (BioLogic Software, Australia, and Kaleida Graph Software, Australia).

### Cell culture and siRNA transfection

MDA-MB-231 cells were purchased from American Type Culture Collection and cultured in EMEM supplemented with 10% fetal bovine serum (FBS), 100 μg/mL streptomycin, 100 units/mL penicillin G at 37 °C in an atmosphere of 5% CO_2_–95% air. siRNA sequences of Nm23-H1, and H2 were previously reported^16^. The following siRNAs were used for Nm23-H1, SiH1: 5′-GGAUUCCGCCUUGUUGGUC and NM23-H2, SiH2: 5′-GGAUUGAUCAUUCUUUUAU. MDA-MB-231 cells were transfected with control or specific 20 nM siRNA by using Lipofectamine RNAiMAX reagent (Thermo Fisher Scientific) according to the manufacturer’s recommendations. Knock down of Nm23-H1 and H2 were verified by anti-Nm23-H1 antibody (Santa Cruz, sc-343), and anti-Nm23-H2 antibody (Thermo Fisher Scientific, PA5–15344), respectively.

### Active Rac1 activity assay

MDA-MB-231 cells were grown in 100 mm dish, and were treated NMac1 or 0.05% DMSO at indicated concentration for 16 h. Active Rac1 pulldown assays were performed according to the manufacturer’s instructions (Thermo Fisher Scientific). Briefly, MDA-MB-231 cell lysates were obtained by scrapping with lysis buffer followed by 8,000 rpm centrifugation. 500 μg of cell lysates were incubates with 20 μg of GST-PBD for 2 h at 4 °C with rocking, and GST-PBD domain interacting with active Rac1 was precipitate with GSH-beads for 1 h at 4 °C with rocking. Precipitates were washed 3 times with lysis buffer, solubilized with gel sample buffer, separated with 12% SDS-PAGE, and detected by Rac1 antibody.

### Immunofluorescence microscopic analysis

MDA-MB-231 cells were grown on the Secureslip^TM^ (Sigma) cell culture glass cover slip to 50% confluence and treated with or without NMac1 for various time. Cells were gently washed with cold HBSS was followed by fixing with 4% paraformaldehyde in HBSS for 10 min at R.T. After washing with HBSS, permeabilization with 0.1% Triton X-100 was performed for 10 min at R.T. Washing with HBSS two times and followed by blocking with 3% BSA, 0.2% Tween20, and 0.2% gelatin in HBSS for 1 h at R.T. F-actin was stained by Rhodamine-Phalloidin (Thermo Fisher Scientific) for 2 h at 37 °C followed by washing 3 times with HBSS for 20 min. Coverslips were mounted with anti-fading solution (Thermo Fisher Scientific), and viewed using a ×63 objective of an LSM510 META (Zeiss) laser scanning confocal microscope. Cell ruffles were quantified by scoring using a scale from 0 to 3, with 0 indicating no protrusion, 1 indicating protrusions in one area of the cell, 2 indicating protrusions in two distinct areas of the cell and 3 indicating protrusions in more than two distinct areas of the cell. The ruffling index was calculated by determining the average of the ruffling scores of 50 cells.

### Invasion and migration assay

Invasion and migration assay were performed using a 24-well Transwell^®^ unit with polycarbonate membrane (pore size, 8 μm) (Corning, USA), with or without Matrigel coating, respectively. The membrane was coated with 50 µg Matrigel^TM^ basement membrane matrix (BD Bioscience, USA). 5 × 10^4^ cells of MDA-MB-231 were seeded into the upper chamber in a serum-free medium with/without NMac1. The lower chamber was filled with a medium containing 10% FBS. After incubation for 24 h at 37 °C, the cells on the upper side of membrane were removed with a cotton swab. The cells invading to the underside of the membrane were stained with 0.5% w/v Crystal violet in 25% methanol and counted at 100-fold magnification under a microscope (Carl Zeiss, Germany). Counted cells were represent to relative values of migrated or invaded cells by NMac1 to control DMSO in three independent triplicate experiments.

### Hydrogen/deuterium exchange mass spectrometry (HDX-MS)

HDX-MS experiment was performed as reported^43^. Nm23-H1 15 μM were incubated with 100 μM NMac1 at R.T. for 30 min in 5% acetonitrile (ACN) followed by incubated with 20-fold D_2_O at 25 °C for the following periods of time: 10, 60, 300, and 1800 s. The deuterium labeling reaction was quenched by 2.5 mM tris (2-carboxyethyl) phosphine (TCEP), formic acid, pH 2.3. For protein digestion, 1 μg of porcine pepsin was added to each quenched protein sample and incubated on ice for 3 min before injection. Peptic peptides were desalted on C_18_ trap column cartridge (Waters) and gradient eluted from 8% ACN to 40% ACN, 0.1% formic acid on 100 µm i.d.× 100 mm analytical column, 1.7 µm particle size, BEH130 C_18_ (Waters) for 7 min. The trap, analytical column and all tubing were immersed in an ice bath to minimize deuterium back-exchange. Gradient chromatography was performed at a flow rate 0.5 μL/min and was sprayed on line to nanoAcquity^TM^/ESI/MS (SYNAPT^TM^ HDMS^TM^) (Waters). The extent of deuterium incorporation was monitored the increase in mass of the isotope distribution for each identified peptide, and calculated by Microsoft Excel and DynamX v3. The theoretical maximum deuterium incorporation value was calculated for each peptide based on the number of exchangeable amides. Each experiment was triplicated.

### Docking of NMac1 and Nm23-H1 by molecular modeling

To predict the binding mode of Nm23-H1 and its activators, NMac1, *in silico* docking studies were performed using the Surflex-Dock program of the SYBYL-X 2.0 software (Tripos, St. Louis, MO, USA). The structures of activators were constructed by the SYBYL sketcher followed by the addition of hydrogen atoms and minimized using the Tripos force field with the Gasteiger–Hückel charge. The monomeric form of Nm23 was used as a template. Addition of hydrogen atoms and assignment of atom charge (MMFF94) were carried out using the structure preparation tool of the SYBYL-X 2.0 software. Prior to *in silico* docking, the coordinates of the template were energy minimized using the Tripos force field. Whole docking was guided by a protomol generated by “residues mode” specifying the mode of construction predicted by HDX analysis according to the software protocol^44,45^. Two important parameters to generate the protomol, ‘Bloat (Å)’, which determines how far the protomol extends into the concavity of the target site, and ‘Threshold’, which impacts on how far the protomol extends outside the concavity, were set to 0.5 and 0, respectively. All other parameters were employed with default settings.

### *In vivo* metastasis assay with NMac1

Mouse study procedures were performed in accordance with protocols approved by the Institutional Animal Care and Use Committee of Seoul National University. 1 × 10^6^ MDA-MB-231-Luc-D3H2LN cells were injected into the mammary fat pad of NOD/SCID mice. NMac1 treatment (10 mg/kg, p.o., daily) was started when the tumors reach 100 mm^3^. Tumor volume was determined by measuring the short and long diameters of the tumors with a caliper. The tumor volume was calculated using the following formula: tumor volume (mm^3^) = (short diameter)^2^ × (long diameter) × 0.5. Metastatic tumor cells were detected by luciferase-based noninvasive bioluminescence imaging using the platform of IVIS Lumina II (Perkin Elmer). According to protocols approved by the Institutional Animal Care and Use Committee of Seoul National University (Approval number; SNU160912-4﻿), mice should be sacrificed when tumor weight exceed 10% of the body weight. In this study, we sacrificed animals when tumor volume exceeds 1500 mm^3^ with monitoring mouse every 2 days.

### WST-1 cell proliferation assay

The cells were dispensed in 96-well, flat-bottom microtiter plates at a density of 2 × 10^3^ cells/well and incubated with NMAC1 at various dilutions for 24 and 48 h followed by an additional 1 h treatment with WST-1 (Roche). The cleavage of WST-1 to formazan by metabolically active cells was quantified by scanning the plates in a microtiter plate reader at 440 and 620 nm (reference wavelength). The test medium was used as the background control. Three independent sets of experiments that were performed in triplicate were evaluated. The viability of the treated cells was normalized to the untreated control cells.

### Statistical analysis

Data were analyzed with Student’s t-test for comparisons between two groups to determine the statistical significance (P value). P < 0.05 was considered to be statistically significant.

### Significance Statement

Tumor metastasis is a major cause of the death of cancer patients. However, there are no therapeutic treatments currently available to prevent this metastasis. Nm23-H1/NDPK-A is firstly identified as a tumor metastasis suppressor having NDP kinase (NDPK) activity, and is positively associated with prolonged disease-free survival and good prognosis of cancer patients. Therefore, it has been thought that overexpression of Nm23-H1 is a good way to inhibit the metastasis. Several approaches were used to increase the cellular level of Nm23-H1 to augment the suppression of tumor metastasis employing gene transfer, protein transduction and glucocorticoid induction. However, these attempts were not practically applicable for the therapy of metastasis. In this study, we found a small molecule that activates Nm23 (NMac1) and identified the mode of action of NMac1. NMac1 suppressed invasion and migration *in vitro* and metastasis *in vivo* in a breast cancer mouse model, and has potential as an anti-metastatic agent.

## Electronic supplementary material

Supplementary Information
