# Supplementary material for: Small molecule activator of Nm23/NDPK as an inhibitor of metastasis
Source: Sci Rep. 2018 Jul 19;8:10909. doi: 10.1038/s41598-018-29101-6 (PMC6053448; doi:10.1038/s41598-018-29101-6)
Supplement: Supplementary file 1 — Supplementary Information [file 41598_2018_29101_MOESM1_ESM.pdf]

Supporting Information

Small molecule activator of Nm23/NDPK as an inhibitor of metastasis

Jae-Jin Lee[1], Hwang Suk Kim[2], Ji Sun Lee[3], Jimin Park[1], Sang Chul Shin[4], Soonwha Song[1], Eunsun Lee[1], Jung-Eun Choi[1], Ji-Wan Suh[1], Hongsoo Lee[2] ,Eunice EunKyeong Kim[4], Eunkyung Seo[1], Dong-Hae Shin[1], Ho-Young Lee[3], Hee-Yoon Lee[2*], Kong-Joo Lee[1,5,*]

[1]Graduate School of Pharmaceutical Sciences, College of Pharmacy, Ewha Womans University, Seoul, 120-750, Korea

[2]Department of Chemistry, Korea Advanced Institute of Science & Technology, Daejeon, 305-701, Korea

[3]College of Pharmacy and Research Institute of Pharmaceutical Sciences, Seoul National University, Seoul 151-742, Korea

[4]Biomedical Research Institute, Korea Institute of Science & Technology, Seoul 02792, Korea

[5]Lead Contact

[*]Correspondence: kjl@ewha.ac.kr, leehy@kaist.ac.k

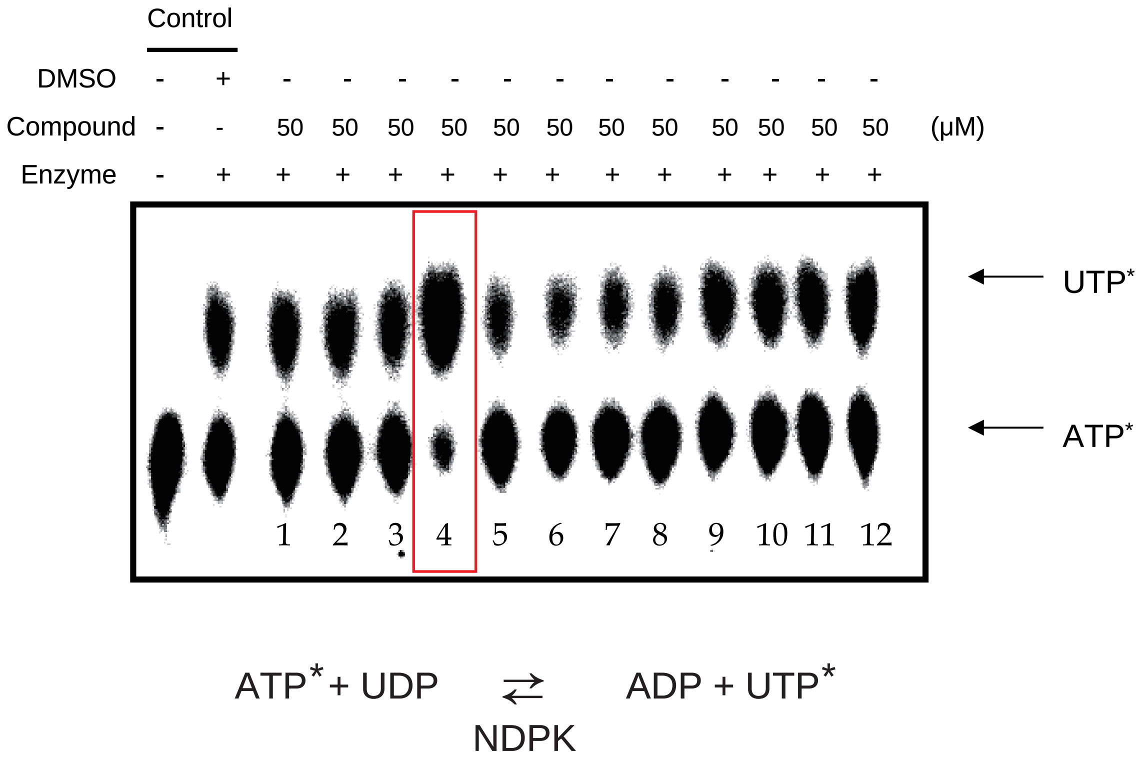

Supplementary Figure 1. NDPK assay with natural product collection.

A screen of the natural compound collection using recombinant Nm23-H1 in NDPK assays that measuring radio-labeled UTP generation from radio-labeled ATP and non-labeled UDP as substrate.

A

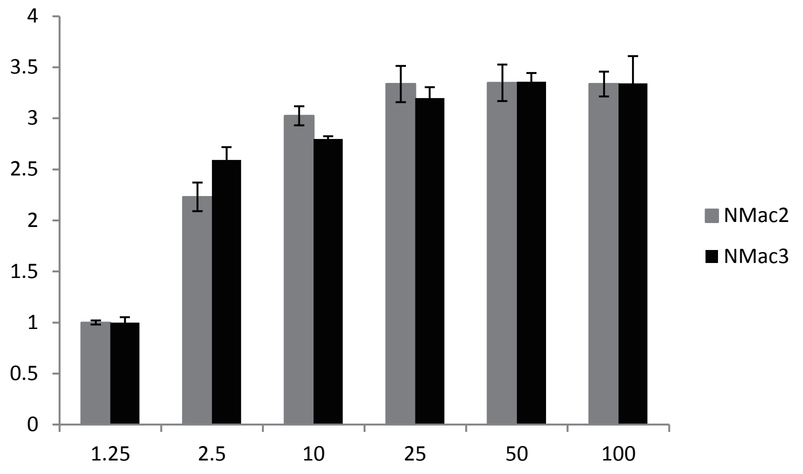

B

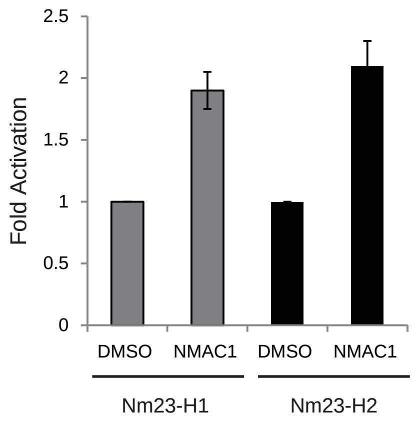

Supplementary Figure 2. Characterization of NDPK activator

(A) The effect of various indicated concentrations of NMac2 and NMac3 on human Nm23-H1 NDPK enzyme activity. NDPK activity was measured by ATP amount that produced by 5 ng of Nm23-H1 using 5 μM ADP and 5 μM UTP for 1 min. All experiments were triplicated, and data are expressed as mean ± s.d. (B) NDPK activation with NMac1 to Nm23-H1 and H2. NDPK activity was measured by ATP amount that produced by 5 ng of Nm23-H1 (or Nm23-H2) using 5 μ M ADP and 5 μM UTP for 1 min. All experiments were triplicated, and data are expressed as mean ± s.d.

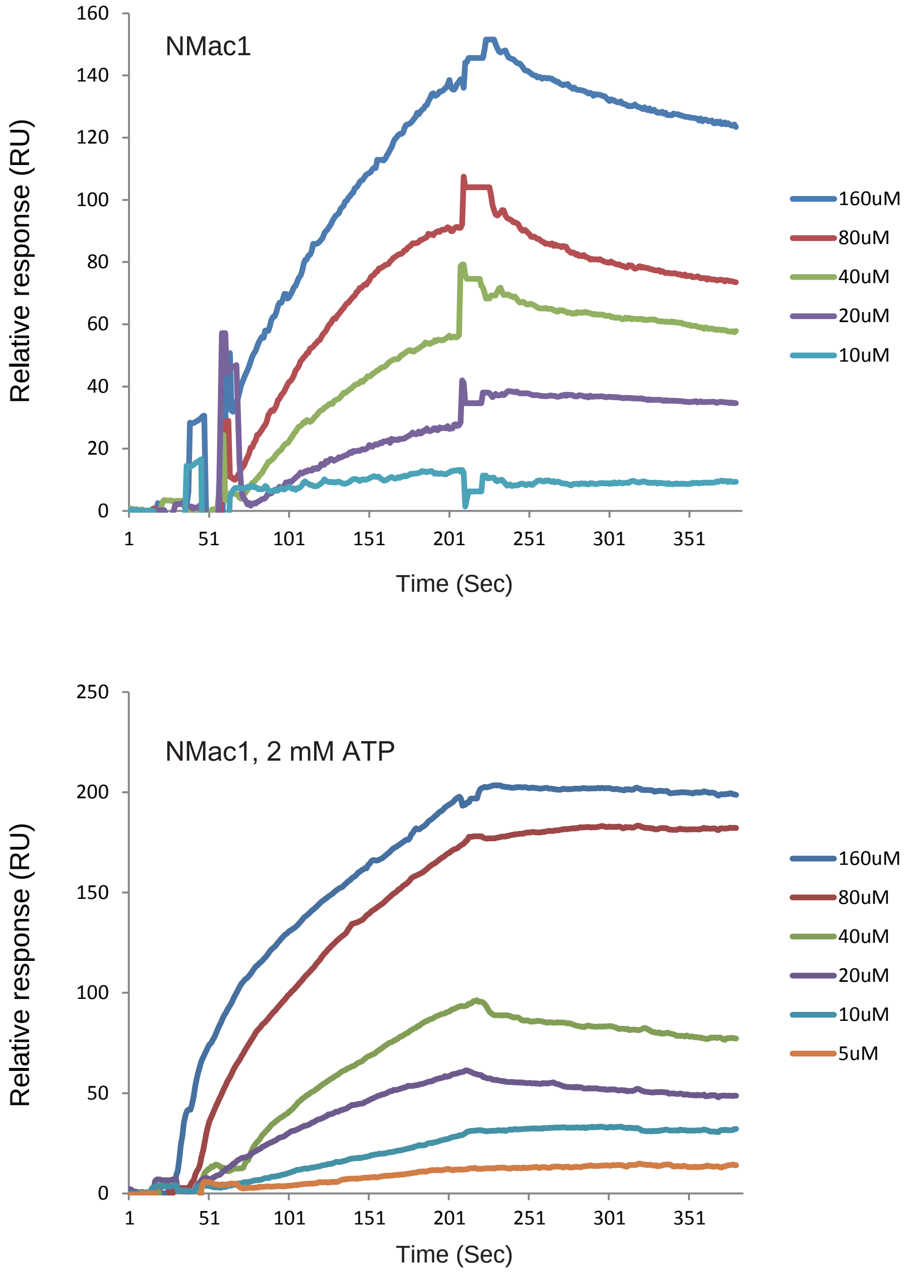

Supplementary Figure 3. Surface plasmon resonance studies for the binding kinetics of Nm23-H1 and NMac1.

The SPR experiments for a simple 1:1 binding model for immobilized Nm23-H1 with NMac1 as the analyte. Pairs of colored traces at each concentration indicate duplicate experimental determinations.

Supplementary Table 1 - Differential deuterium exchange rates of identified Nm23-H1 peptides in HDX-MS experiment.

Sequence	Start	End	Differential deuterium exchange rate (%)				
			10 sec	60 sec	300 sec	1800 sec	mean
ANCERTF	2	8	-14.3	-14.3	0.0	0.0	-7.1
ERTFIAIKPDGVQRGLVGE	5	23	0.0	0.0	0.0	0.0	0.0
IAIKPDGVQRGLVGE	9	23	0.0	0.0	0.0	0.0	0.0
IAIKPDGVQRGLVGEIIKRFEQKGFRL	9	35	0.0	0.0	0.0	0.0	0.0
VGLKFMQASEDLLKEHYVDL	36	55	0.0	0.0	0.0	0.0	0.0
MQASEDLLKEHYVDLKDRPFFAGL	41	64	0.0	0.0	0.0	0.0	0.0
LKEHYVDLKDRPFFAG	48	63	0.0	0.0	6.7	6.7	3.3
LVKYMHSGPVVA	64	75	-9.1	-4.5	-4.5	-4.5	-5.7
MVWEGLN	76	82	0.0	0.0	0.0	0.0	0.0
NVVKTGRVML	82	91	0.0	0.0	0.0	0.0	0.0
VVKTGRVML	83	91	-11.1	0.0	0.0	0.0	-2.8
GETNPADSKPGTIRGDF	92	108	-3.3	-3.3	-3.3	-3.3	-3.3
CIQVGRNIIHGSD	109	121	-3.8	-3.8	0.0	0.0	-1.9
CIQVGRNIIHGSDSVESAEKEIGL	109	132	0.0	0.0	0.0	0.0	0.0
WFHPEELVD	133	141	0.0	0.0	0.0	0.0	0.0
YTSCAQNWIYE	142	152	-9.1	-13.6	-9.1	-9.1	-10.2

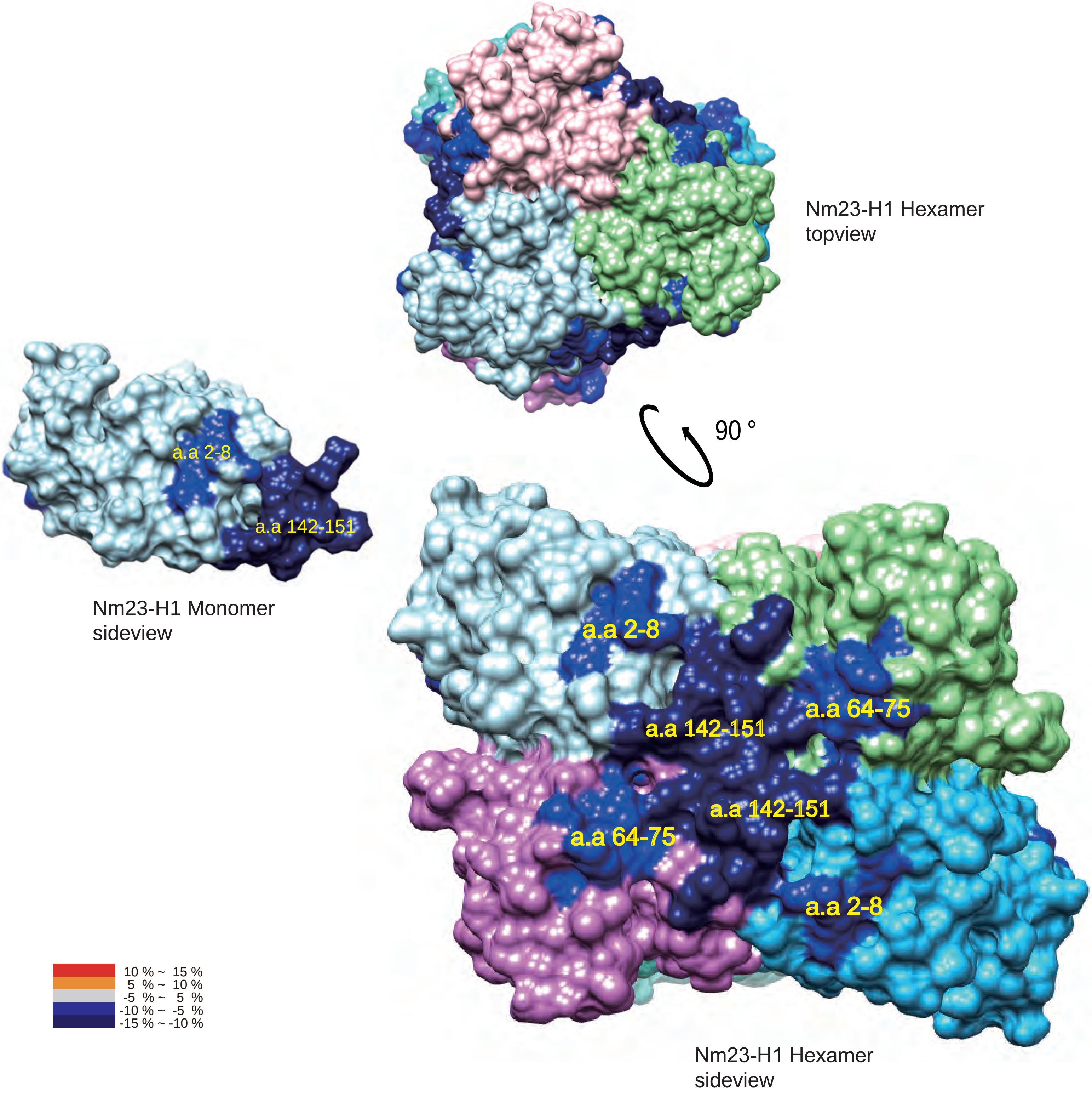

Supplementary Figure 4. Overlay of differential HDX data of NMac1 bound to Nm23-H1 hexamer.

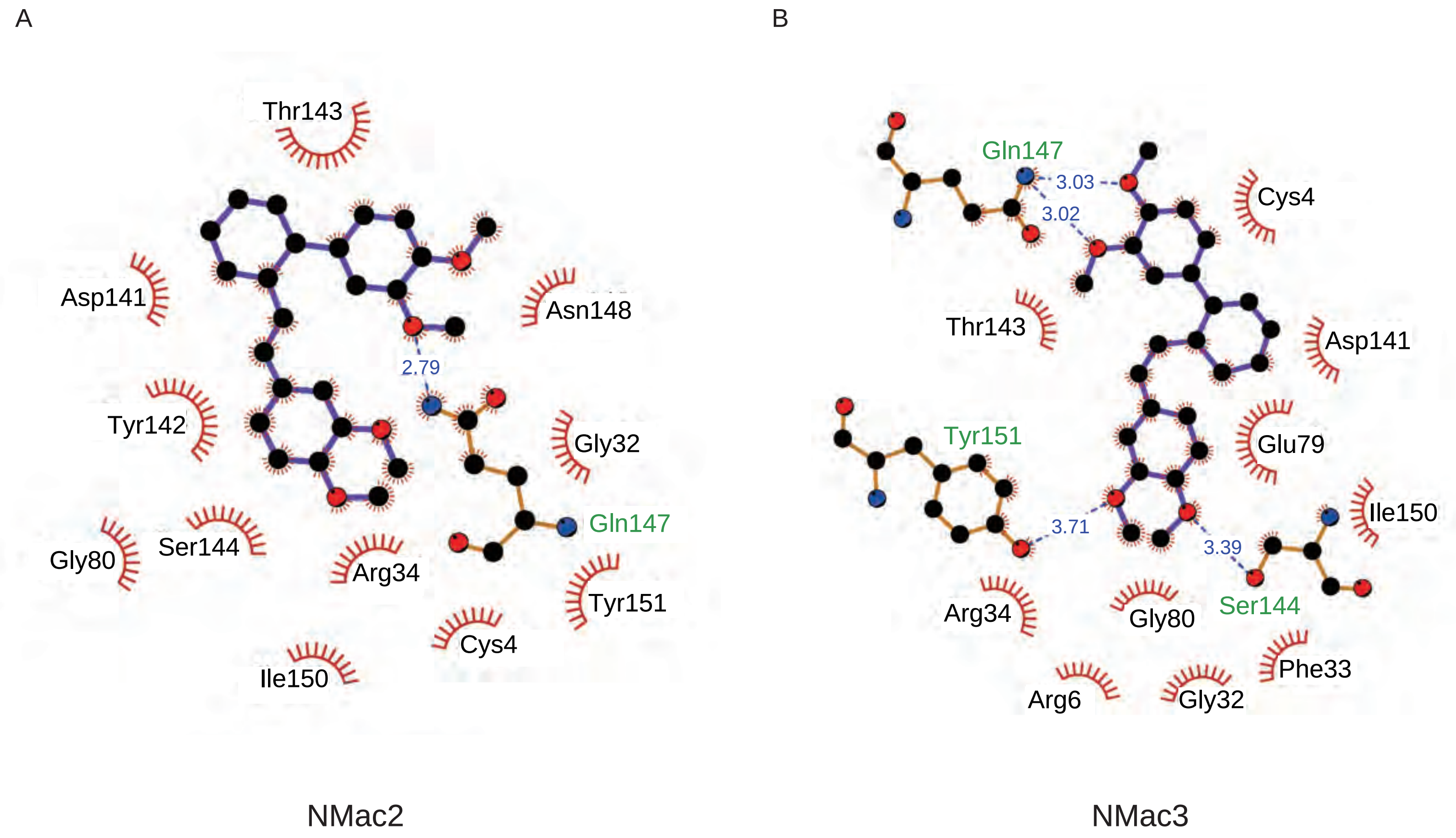

Supplementary Figure 5. 2D plot of the binding modes was generated by Ligplot plus.

2D plot of the binding modes of NMac2 (A), and NMac3 (B). Hydrophobic contacts are depicted by an arc with spokes radiating towards the ligand atoms they contact. Hydrogen bonds are indicated by dashed lines between the atoms involved and their lengths (Å) are labeled. The carbon, oxygen and nitrogen atoms are colored in black, red and blue, respectively.

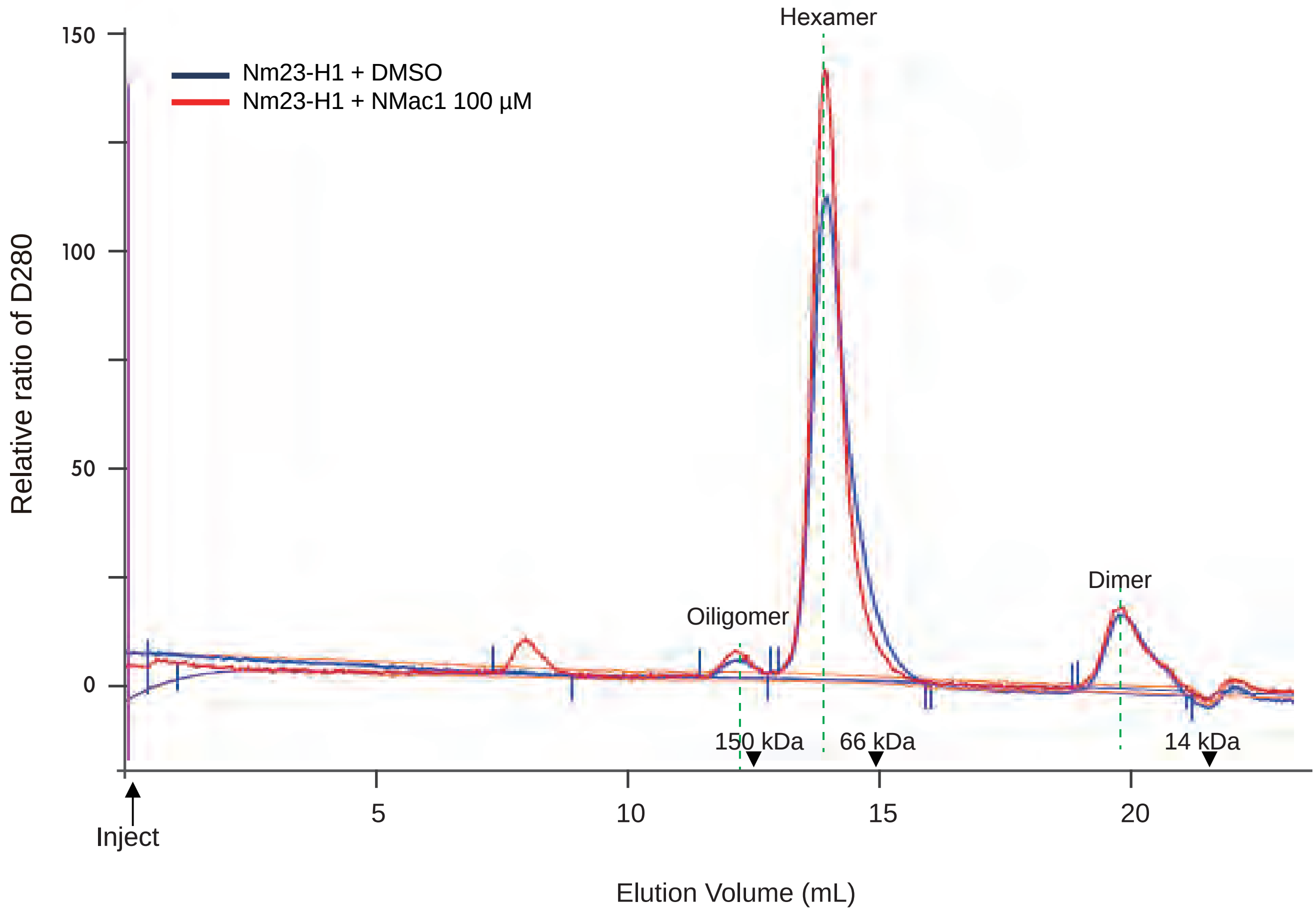

Supplementary Figure 6. NMac1 does not affect on hexamer formation of Nm23-H1

Size exclusion chromatograms of Nm23-H1 protein treated with 100 μM NMac1; Red line (or 2% DMSO; Blue line). 100 μg of Nm23-H1 was treated with 100 μM NMac1 or 2% DMSO as vehicle for 10 min at R.T followed by size exclusion chromatography with elution buffer (20 mM HEPES, 3 mM $MgCl_2$). Alcohol dehydrogenase (150 kDa) Albumin (66 kDa) and α-lactalbumin (14 kDa) were used as molecular weight markers.

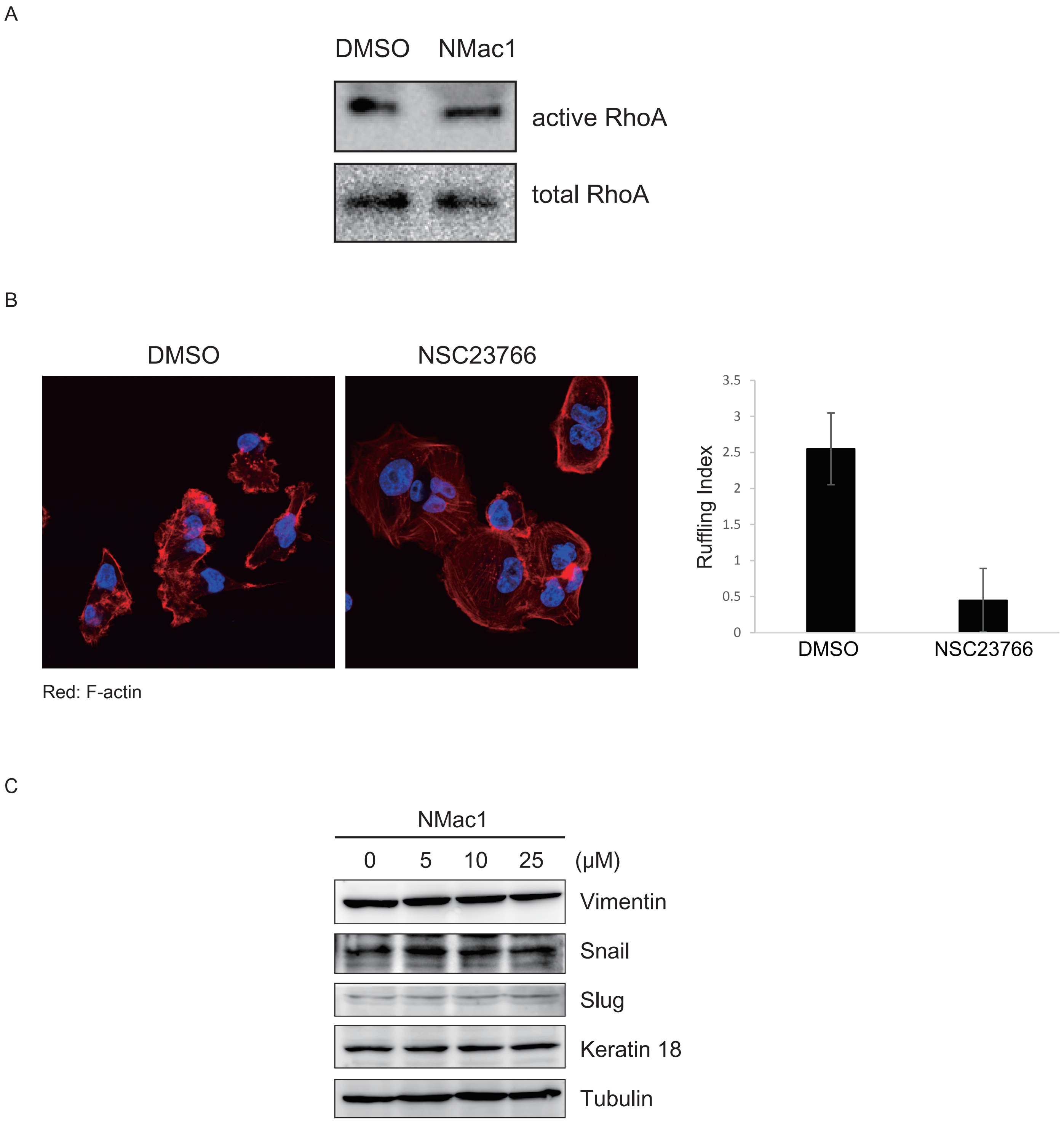

Supplementary Figure 7. NMac1 induced morphological chage of MDA-MB-231 cell through Rac1 inactivation.

(A) Active RhoA pulldown assay was conducted in control and 25 μM NMac1 treated MDA-MB-231 cells for 16 h. (B) Rac1 inhibitor shows similar mophological change to NMac1 in MDA-MB-231 cells. Localization of F-actin was analyzed by confocal microscopy. MDA-MB-231 cells treated NSC23766 50 μM (or 0.1% DMSO as vehicle) for 4 h were stained by phalloidin-Rhodamine. Cell ruffling of MDA-MB-231 cells was quantified by determining the ruffling index (C) EMT markers were detected with anti-vimentin, snail, slug, and keratin 18 antibodies, and Tubulin were detected to varify amount of loaded total proteins.

A

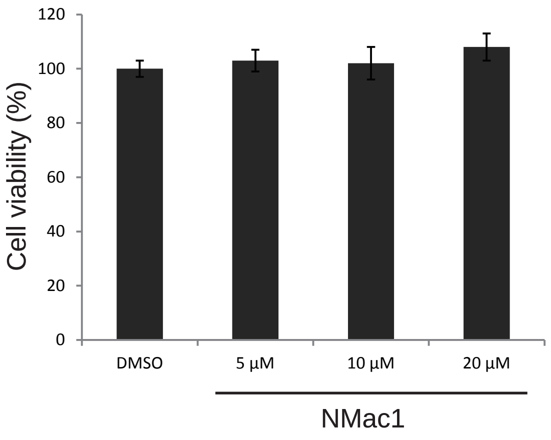

B

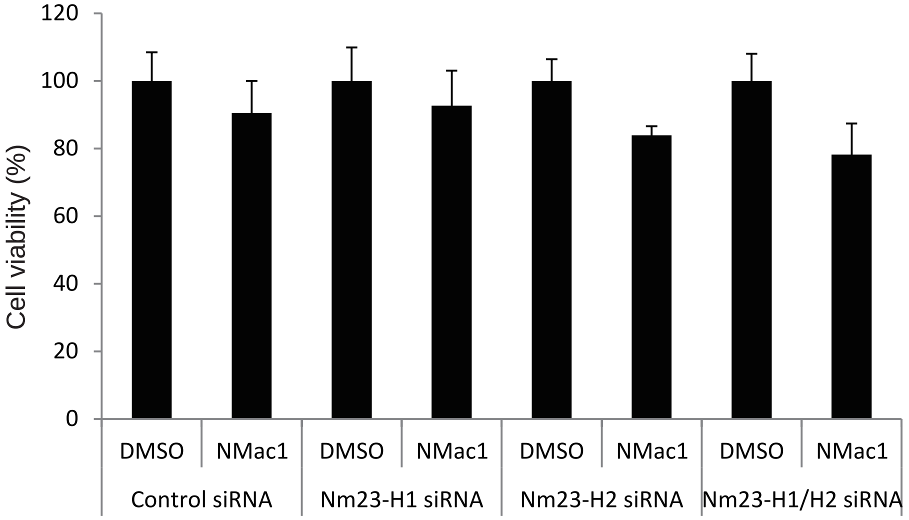

Supplementary Figure 8. WST-1 Proliferation assay

(A) MDA-MB-231 cells were treated with indicated concentrations of NMac1 for 48 h in serum free media, followed by cell proliferations assay with WST-1 reagent. (B) Nm23-H1 (or/and) H2 knocked down MDA-MB-231 cells were treated with 25 μM NMac1 for 48 h in serum free media, followed by cell proliferations assay with WST-1 reagent.

A

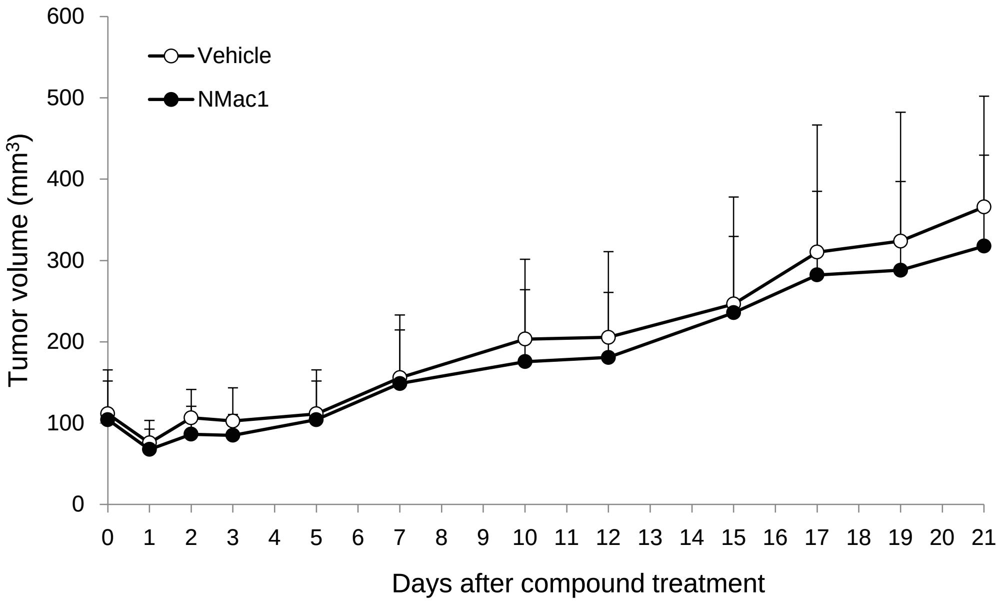

B

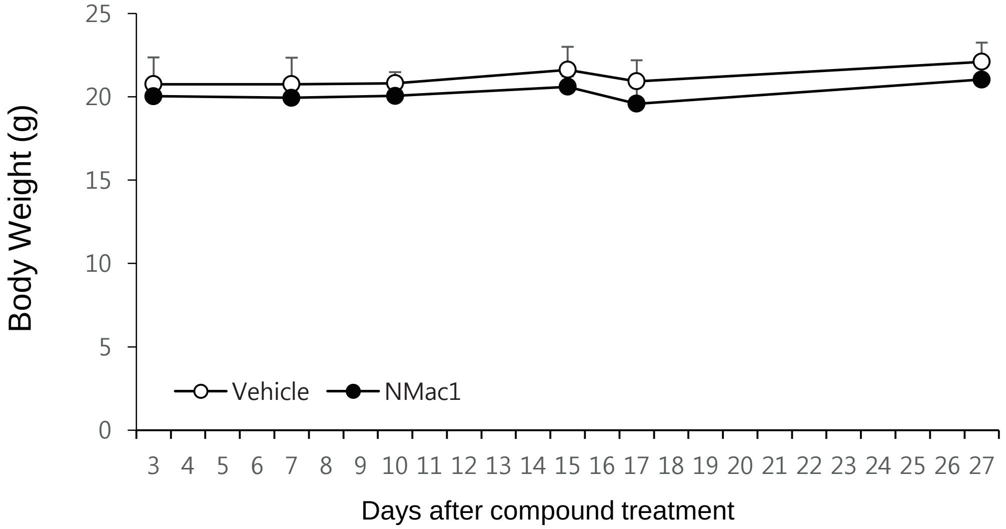

Supplementary Figure 9. Primary tumor size and body weight of Vehicle or NMac1 treated mice

MDA-MB-231-Luc-D3H2LN cells were orthotopically injected into NOD/SCID mice and the mice were treated with vehicle or NMac1 (n=7 for VEH group, n=6 for NMac1 group). Primary tumor size (A) and body weight (B) were assessed 3 weeks after the NMac1 treatment. Data are presented as mean tumor volume (or body weight) ± S.D.

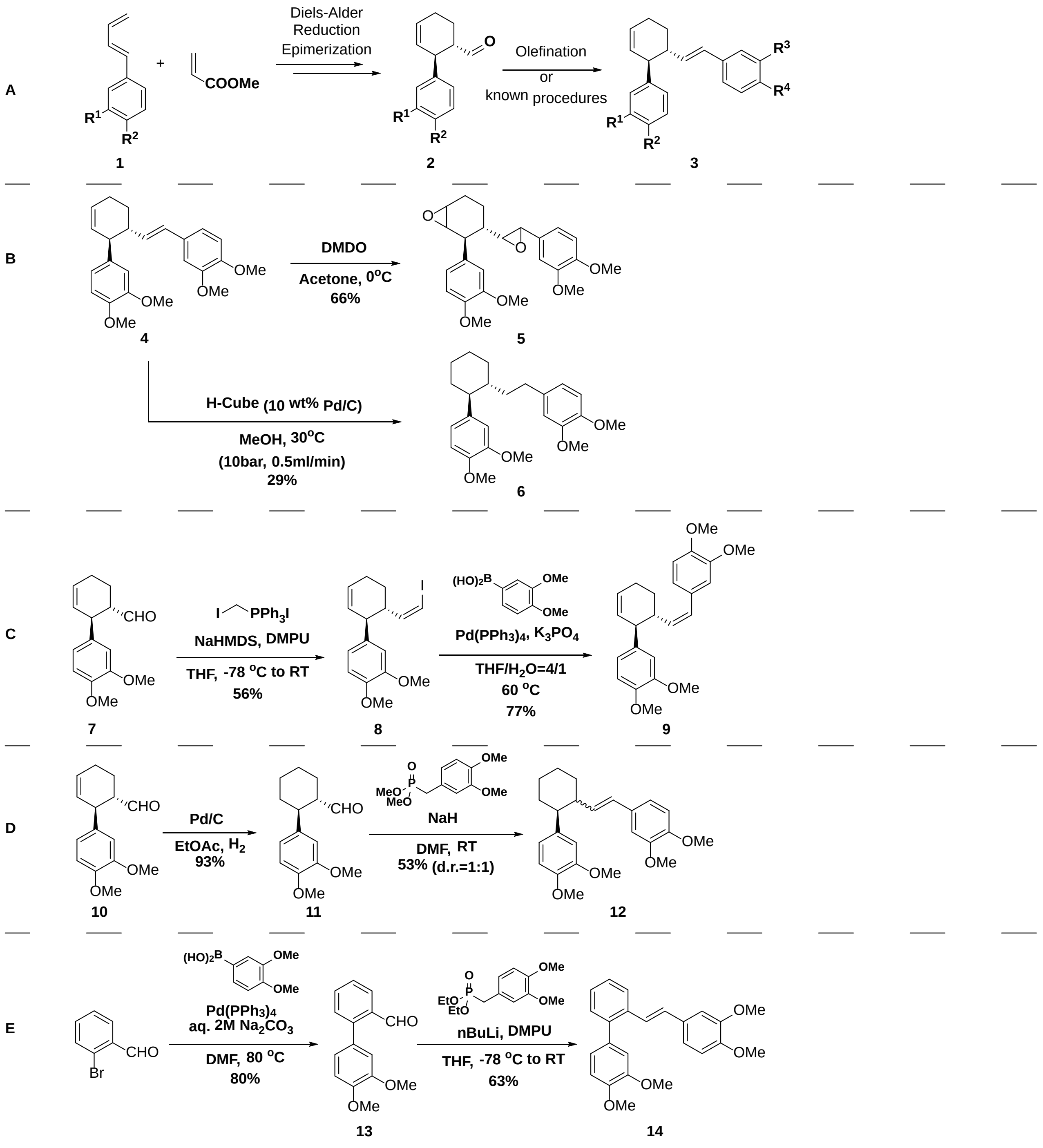

Supplementary Figure 10. Synthesis of NMac1 derivatives

A

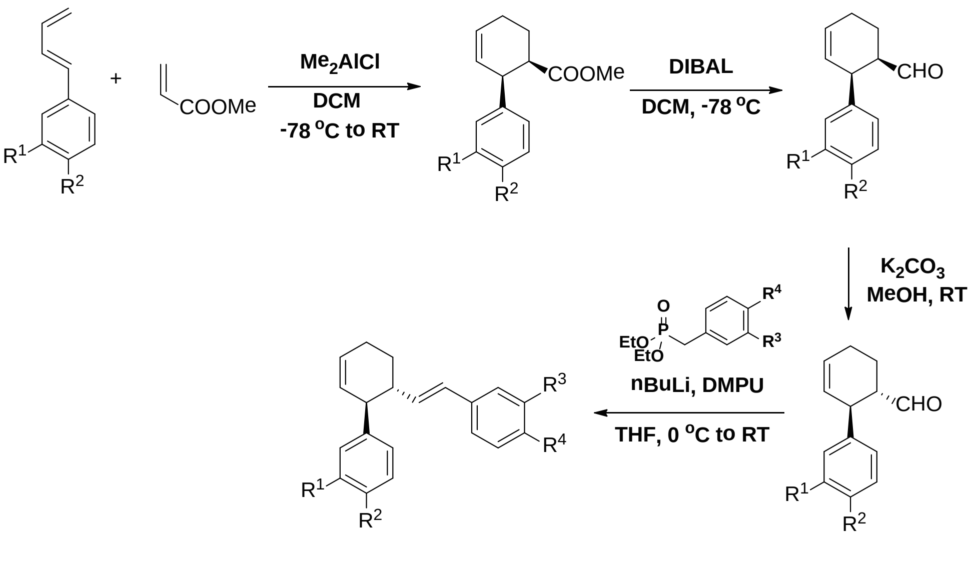

B

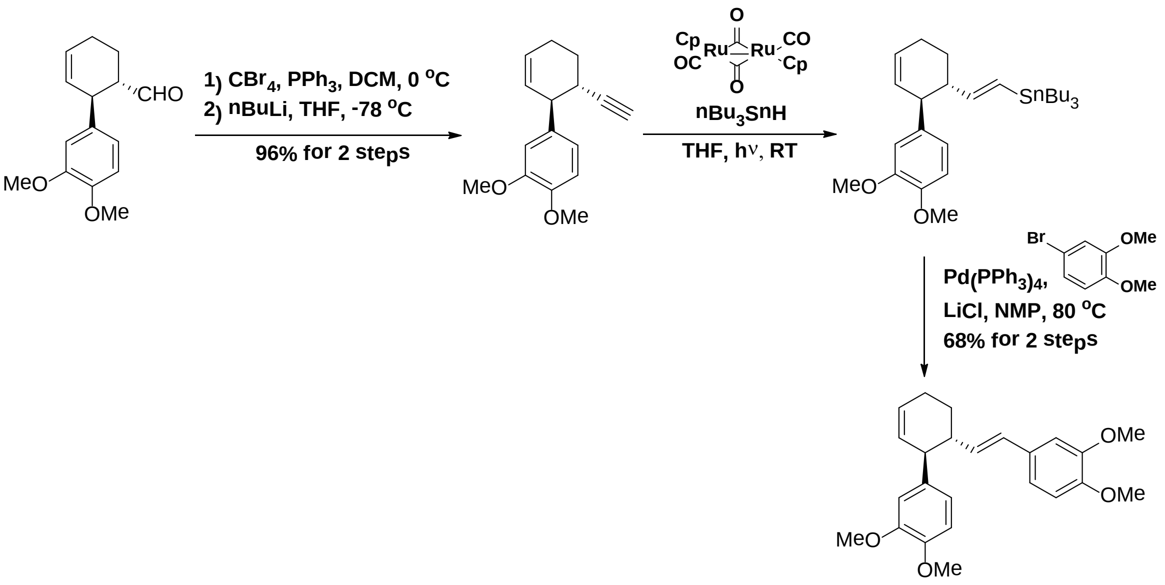

Supplementary Figure 11. (A) General procedures for NMac1 derivatives (B) Alternative procedures for NMac1

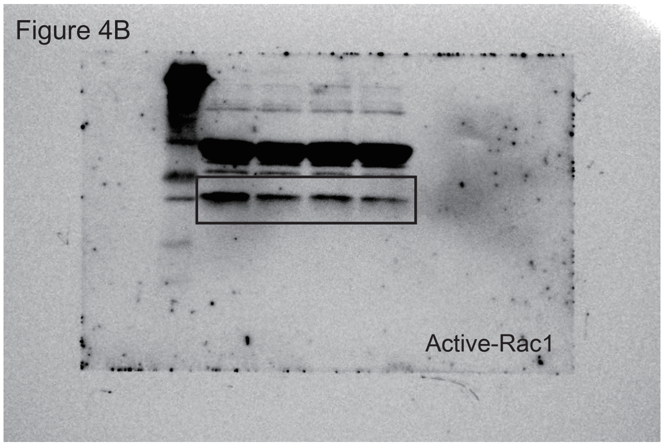

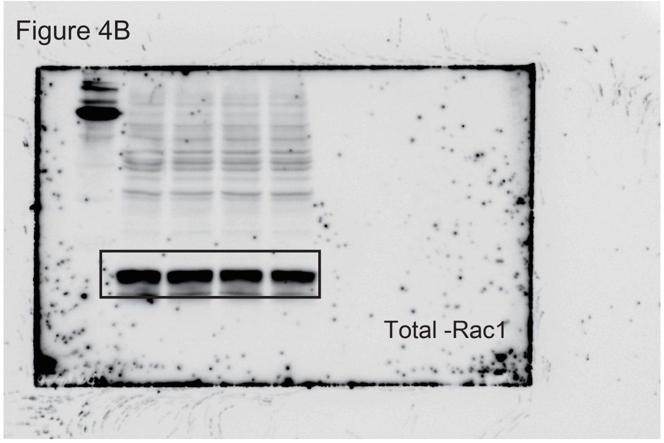

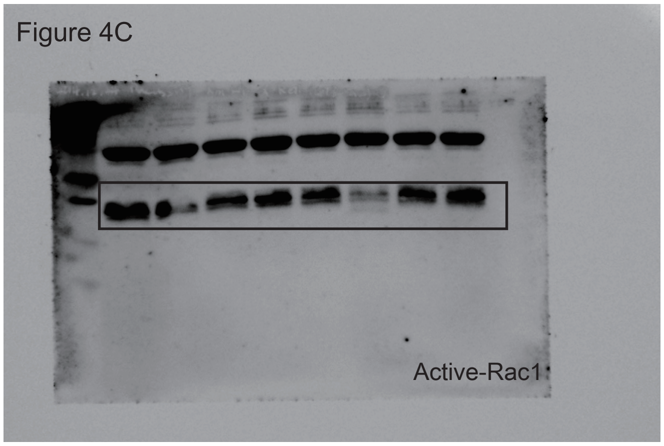

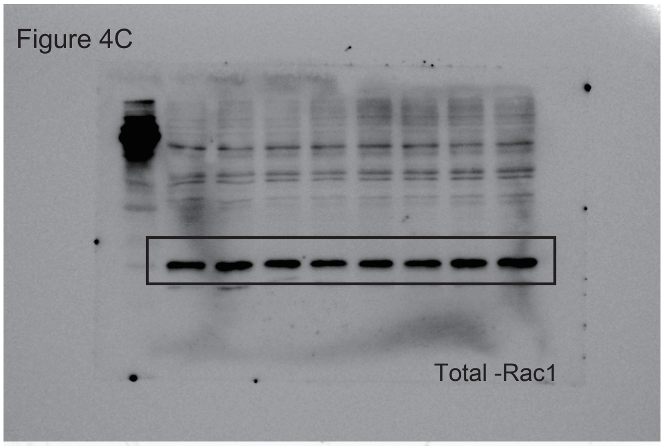

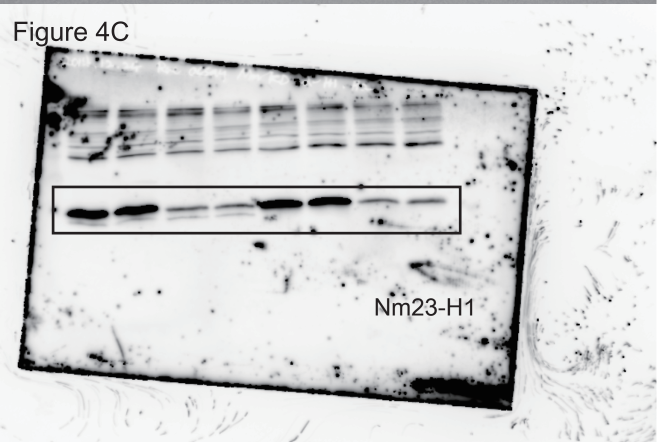

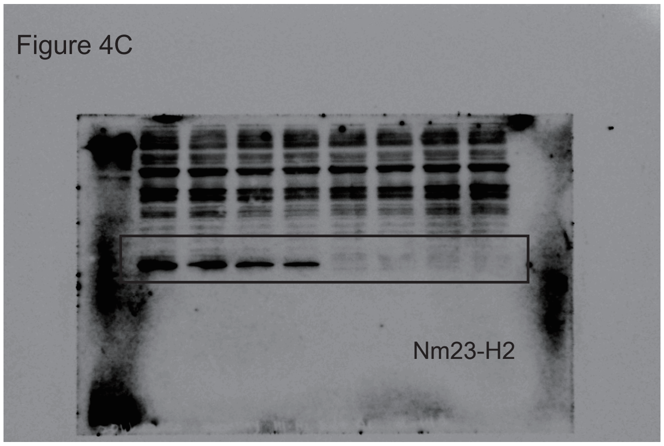

Supplementary Figure 12 - Full size gels used in Figure 4

1. General information

1.1 General procedures

All oxygen- or moisture-sensitive reactions were carried out in oven-dried glassware under a positive pressure of argon. Sensitive liquids and solutions were transferred by syringe or cannula and were introduced through rubber septa through which a high flow of inert gas was maintained. Unless otherwise stated, reactions were carried out at room temperature. Concentration of solutions was accomplished using a Buchi rotary evaporator with an air aspirator. This was generally followed by removal of residual solvents on a vacuum line held at 0.1-1 torr.

1.2 Reagents and solvents

Unless otherwise noted, all reagents and solvents were used without additional purification. Exceptions include: chromatography grade hexane and ethyl acetate used technical grade and distilled before use; Et_2O and THF were distilled from sodiumbenzophenone ketyl under nitrogen; triethylamine were distilled from sodium; dichloromethane was distilled from P_2O_5. Concentration of alkyllithium solutions was determined by titration against diphenylacetic acid. Other reagents or transition metal catalysts were used from purchased commercial source such as Sigma-Aldrich and TCI.

1.3 Chromatography

Analytical thin layer chromatography (TLC) was performed on Merck precoated Analytical thin layer chromatography (TLC) was performed on Merck precoated silica gel 60 F254 plates. Visualization on TLC was achieved by use of UV light (254 nm), exposure to iodine vapor, or treatment with acidic anisaldehyde or ceric ammonium molybdate stain followed by heating. Flash column chromatography was carried out using Merck 60, 230-400 mesh ASTM.

1.4 Physical and spectroscopic measurements

Proton-1 nuclear magnetic resonance spectroscopy (1H NMR) was recorded on Bruker Fourier Transform AV300 (300 MHz) spectrometers, Bruker Fourier Transform AV400 (400MHz) spectrometers or Agilent Technologies DD2 (600 MHz). Chemical shifts were reported in δ units, parts per million (ppm) relative to the singlet as 7.24 ppm for chloroform-d. The following abbreviations were used to describe peak patterns when appropriate: b = broad, s = singlet, d = doublet, t = triplet, q = quartet, m = multiplet. Coupling constant, J, was reported in Hertz unit (Hz). Carbon-13 nuclear magnetic resonance spectroscopy (13C NMR) was recorded on Bruker Fourier Transform AM 400 (100 MHz) or Agilent Technologies DD2 (150 MHz) and was fully decoupled by broad-band decoupling. Chemical shifts were reported in ppm with the centerline of the triplet for chloroform-d set at 77.00 ppm.

2. Experimental procedures and spectral data

General Procedure A: Diels-Alder reaction of 1,3-diene and methyl acrylate

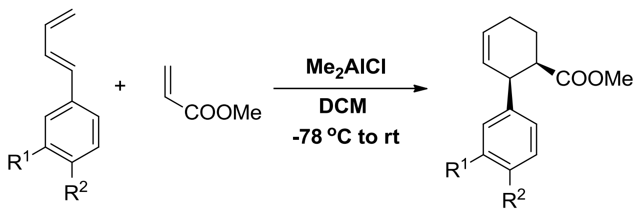

To a stirred solution of 1,3-diene(1.0 eq.) and methyl acrylate(1.5 eq.) in DCM was added Me_2AlCl(1.0M in Hx, 1.1eq.) slowly at -78 °C under Ar. The resulting mixture was allowed to warm up to room temperature and stirred for overnight. The solution was cooled down to 0 °C and quenched with aq. NH_4Cl solution and distilled water. Extracted with dichloromethane and dried over $MgSO_4$. Filtered it and concentrated *in vacuo*. The residue was purified by flash column chromatography on silica gel.

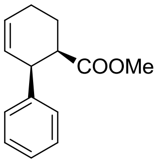

##

was prepared with General Procedure A to give two diastereomers in the ratio of *cis/trans*=10/1. 70% Yield;

^{1}H NMR (400 MHz, $CDCl_3$) δ 7.34 – 7.25 (m, 2H), 7.28 – 7.17 (m, 3H), 6.00 (ddt, *J* = 9.4, 4.4, 1.8 Hz, 1H), 5.78 (dddd, *J* = 9.9, 4.6, 2.6, 1.7 Hz, 1H), 3.90 (td, *J* = 5.4, 2.6 Hz, 1H), 3.51 (s, 3H), 3.03 – 2.92 (m, 1H), 2.38 – 2.25 (m, 1H), 2.25 – 2.10 (m, 1H), 1.90 – 1.77 (m, 2H).

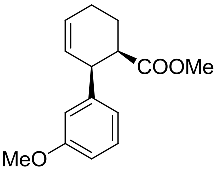

##

was prepared with General Procedure A to give two diastereomers in the ratio of *cis/trans*=10/1. 69% Yield;

^{1}H NMR (400 MHz, $CDCl_3$) δ 7.16 (td, *J* = 7.5, 1.1 Hz, 1H), 6.78 – 6.70 (m, 3H), 5.94 (ddt, *J* = 9.3, 4.2, 1.6 Hz, 1H), 5.72 (dddd, *J* = 9.8, 4.6, 2.6, 1.7 Hz, 1H), 3.83 (td, *J* = 4.9, 2.4 Hz, 1H), 3.75 (s, 3H), 3.49 (s, 3H), 2.91 (ddd, *J* = 11.8, 5.9, 3.9 Hz, 1H), 2.32 – 2.19 (m, 1H), 2.20 – 2.04 (m, 1H), 1.86 – 1.73 (m, 2H).

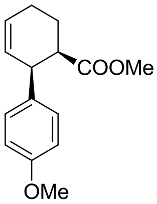

##

was prepared with General Procedure A to give two diastereomers in the ratio of *cis/trans*=10/1. 92% Yield;

^{1}H NMR (400 MHz, , $CDCl_3$) δ 7.07 (d, *J* = 8.5 Hz, 2H), 6.78 (d, *J* = 8.8 Hz, 2H), 5.92 (dtd, *J* = 9.1, 4.3, 2.0 Hz, 1H), 5.71 (ddq, *J* = 9.8, 4.6, 1.7 Hz, 1H), 3.80 (tq, *J* = 5.5, 2.9 Hz, 1H), 3.75 (s, 3H), 3.48 (s, 3H), 2.93 – 2.83 (m, 1H), 2.31 – 2.18 (m, 1H), 2.20 – 2.04 (m, 1H), 1.82 – 1.71 (m, 2H).

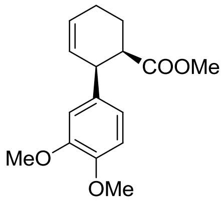

##

was prepared with General Procedure A to give two diastereomers in the ratio of *cis/trans*=14/1. 49% Yield;

^{1}H NMR (400 MHz, $CDCl_3$) δ 6.75 (d, *J* = 8.0 Hz, 1H), 6.71 (d, *J* = 2.0 Hz, 1H), 6.71 – 6.65 (m, 1H), 5.92 (dtd, *J* = 9.1, 4.4, 2.2 Hz, 1H), 5.76 – 5.66 (m, 1H), 3.82 (s, 6H), 3.82 – 3.78 (m, 1H), 3.48 (s, 3H), 2.93 – 2.83 (m, 1H), 2.30 – 2.18 (m, 1H), 2.18 – 2.03 (m, 1H), 1.81 – 1.71 (m, 2H).

General Procedure B: DIBAL reduction of ester to aldehyde

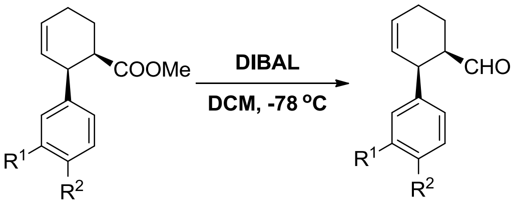

To a stirred solution of methyl ester(1.0 eq.) in DCM was added DIBAL(1.0M in DCM, 1.05 eq.) slowly at -78 °C under Ar. The resulting mixture was stirred for 2h at -78°C. Then H_2O(10 eq.) and NaF(5.0 eq.) were added at -78 °C and stirred for 30min at room temperature. The mixture was filtered through silica/Celite pad and washed with dichloromethane. The filtrate was concentrated *in vacuo* and the residue was purified by flash column chromatography on silica gel.

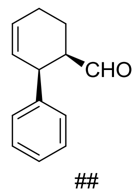

##

Prepared from ## with General Procedure B. 96% Yield;

^{1}H NMR (400 MHz, $CDCl_3$) δ 9.49 (d, *J* = 1.8 Hz, 1H), 7.32 – 7.24 (m, 2H), 7.26 – 7.16 (m, 3H), 5.98 (dddd, *J* = 10.0, 4.5, 2.9, 1.9 Hz, 1H), 5.82 (dddd, *J* = 10.0, 4.3, 2.4, 1.9 Hz, 1H), 3.96 (ddq, *J* = 6.0, 4.1, 1.9 Hz, 1H), 2.81 – 2.71 (m, 1H), 2.35 – 2.21 (m, 1H), 2.21 – 2.07 (m, 1H), 1.91 – 1.80 (m, 2H).

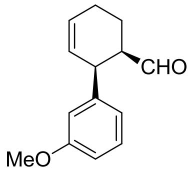

##

Prepared from ## with General Procedure B. 86% Yield;

^{1}H NMR (400 MHz, $CDCl_3$) δ 9.49 (d, *J* = 2.0 Hz, 1H), 7.24 – 7.15 (m, 1H), 6.82 – 6.73 (m, 3H), 5.97 (dtd, *J* = 11.9, 2.9, 2.0 Hz, 1H), 5.81 (ddt, *J* = 10.0, 4.3, 2.2 Hz, 1H), 3.93 (ddq, *J* = 6.1, 4.1, 2.2 Hz, 1H), 3.76 (s, 3H), 2.74 (ddt, *J* = 8.4, 6.0, 2.9 Hz, 1H), 2.32 – 2.20 (m, 1H), 2.13 (dddq, *J* = 18.3, 7.8, 5.2, 2.7 Hz, 1H), 1.91 – 1.81

(m, 2H).

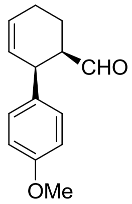

##

Prepared from ## with General Procedure B. 73% Yield;

^{1}H NMR (400 MHz, $CDCl_3$) δ 9.49 (d, *J* = 2.1 Hz, 1H), 7.11 (d, *J* = 8.8 Hz, 2H), 6.81 (d, *J* = 8.8 Hz, 2H), 5.95 (dddd, *J* = 10.1, 4.4, 2.7, 1.9 Hz, 1H), 5.78 (dddd, *J* = 10.0, 4.4, 2.5, 1.9 Hz, 1H), 3.91 (dddd, *J* = 6.3, 4.1, 2.5, 1.2 Hz, 1H), 3.76 (s, 3H), 2.72 (dddd, *J* = 10.1, 5.7, 4.5, 2.0 Hz, 1H), 2.26 (dddt, *J* = 15.9, 7.3, 4.2, 1.7 Hz, 1H), 2.13 (dddd, *J* = 18.4, 9.4, 6.7, 2.7 Hz, 1H), 1.87 – 1.76 (m, 2H).

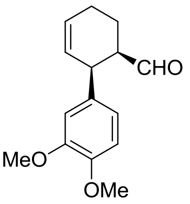

##

Prepared from ## with General Procedure B. 89% Yield;

^{1}H NMR (400 MHz, $CDCl_3$) δ 9.46 (d, *J* = 2.1 Hz, 1H), 6.78 – 6.70 (m, 2H), 6.68 (d, *J* = 2.0 Hz, 1H), 5.94 (dddd, *J* = 10.2, 4.6, 2.9, 1.9 Hz, 1H), 5.77 (ddq, *J* = 11.2, 4.5, 2.3 Hz, 1H), 3.88 (dtd, *J* = 8.4, 4.2, 1.8 Hz, 1H), 3.81 (s, 3H), 3.80 (s, 3H), 2.74 – 2.65 (m, 1H), 2.30 – 2.19 (m, 1H), 2.11 (dddt, *J* = 15.6, 10.3, 5.1, 2.7 Hz, 1H), 1.82 (ddt, *J* = 7.2, 5.6, 3.9 Hz, 2H).

General Procedure C: Epimerization

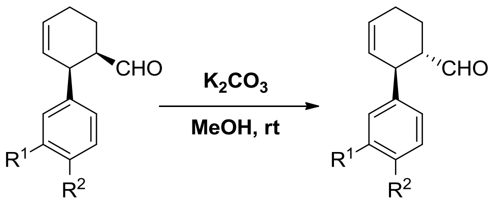

To a stirred solution of *cis*-aldehyde(1.0 eq.) in MeOH was added K_2CO_3(1.3 eq.) at room temperature under Ar. The resulting mixture was stirred for 36 - 48h. Then the mixture was diluted with ethyl acetate and quenched with aq. NH_4Cl solution and distilled water. Extracted with ethyl acetate and dried over $MgSO_4$. Filtered it and concentrated *in vacuo.* The residue was purified by flash column chromatography on silica gel.

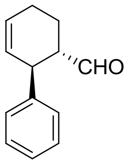

##

was prepared by General procedure C, Yield = 64%;

^{1}H NMR (599 MHz, $CDCl_3$) δ 9.67 (d, *J* = 1.3 Hz, 1H), 7.29 (dd, *J* = 8.3, 6.9 Hz, 2H), 7.24 – 7.17 (m, 3H), 5.89 (dq, *J* = 9.8, 3.7 Hz, 1H), 5.67 (dq, *J* = 10.0, 2.2 Hz, 1H), 3.76 (dp, *J* = 7.7, 2.5 Hz, 1H), 2.59 (dddd, *J* = 9.1, 7.5, 3.4, 1.4 Hz, 1H), 2.17 (ddd, *J* = 6.0, 4.7, 2.7 Hz, 2H), 1.94 (dtd, *J* = 14.5, 5.6, 3.6 Hz, 1H), 1.74 (dddd, *J* = 13.7, 9.3, 7.7, 6.4 Hz, 1H).

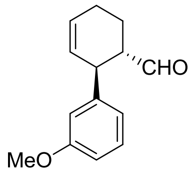

##

was prepared by General procedure C, Yield = 50%;

^{1}H NMR (400 MHz, $CDCl_3$) δ 9.68 (d, *J* = 1.5 Hz, 1H), 7.21 (td, *J* = 7.5, 1.1 Hz, 1H), 6.81 (dtd, *J* = 7.5, 1.1, 0.4 Hz, 1H), 6.78 – 6.72 (m, 2H), 5.88 (dtd, *J* = 9.8, 3.7, 2.2 Hz, 1H), 5.66 (ddt, *J* = 10.0, 3.2, 2.2 Hz, 1H), 3.78 (s, 3H), 3.75 – 3.71 (m, 1H), 2.58 (dddd, *J* = 9.2, 7.4, 3.5, 1.5 Hz, 1H), 2.16 (dtd, *J* = 8.6, 6.2, 3.4 Hz, 2H), 1.98 – 1.89 (m, 1H), 1.79 – 1.69 (m, 1H).

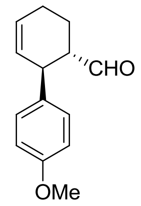

##

was prepared by General procedure C, Yield = 50%;

^{1}H NMR (400 MHz, $CDCl_3$) δ 9.66 (d, *J* = 1.6 Hz, 1H), 7.13 (d, *J* = 8.8 Hz, 2H), 6.83 (d, *J* = 8.8 Hz, 2H), 5.86 (dtd, *J* = 9.7, 3.6, 2.3 Hz, 1H), 5.64 (ddt, *J* = 10.0, 3.1, 2.2 Hz, 1H), 3.77 (s, 3H), 3.70 (dp, *J* = 7.9, 2.7 Hz, 1H), 2.54 (dddd, *J* = 9.3, 7.5, 3.5, 1.6 Hz, 1H), 2.20 – 2.12 (m, 2H), 1.93 (dtd, *J* = 13.4, 5.5, 3.5 Hz, 1H), 1.72 (dddd, *J* = 13.4, 9.5, 7.6, 6.6 Hz, 1H).

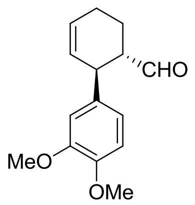

##

was prepared by General procedure C, Yield = 77%;

^{1}H NMR (400 MHz, $CDCl_3$) δ 9.67 (d, *J* = 1.6 Hz, 1H), 6.78 (d, *J* = 8.2 Hz, 1H), 6.75 (dd, *J* = 8.2, 1.9 Hz, 1H), 6.71 (d, *J* = 1.9 Hz, 1H), 5.87 (dtd, *J* = 9.8, 3.7, 2.3 Hz, 1H), 5.66 (ddt, *J* = 10.0, 3.0, 2.1 Hz, 1H), 3.85 (s, 3H), 3.83 (s, 3H), 3.69 (dp, *J* = 8.0, 2.8 Hz, 1H), 2.56 (dddd, *J* = 9.3, 7.6, 3.4, 1.6 Hz, 1H), 2.16 (dtd, *J* = 5.1, 3.1, 1.5 Hz, 2H), 1.93 (dtd, *J* = 14.4, 5.5, 3.4 Hz, 1H), 1.73 (dddd, *J* = 13.4, 9.5, 7.8, 6.4 Hz, 1H).

General Procedure D: Horner-Wadworths-Emmons olefination

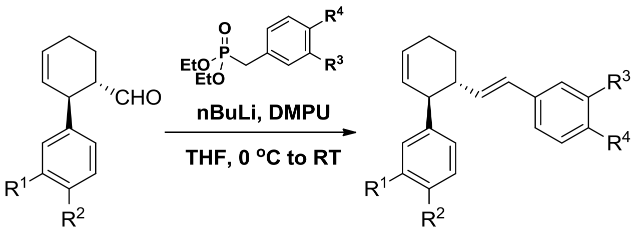

To a stirred solution of dialkyl benzylphosphonate(2.0 eq.) in THF was added nBuLi(2.48M in Hx, 1.95 eq.) at 0 °C under Ar. The resulting mixture was stirred for 30min at room temperature and cooled down again to 0 °C. The solution of *cis*-aldehdye(1.0 eq.) and DMPU(5.0 eq.) in THF was added to the prepared ylide solution by cannula under Ar. The resulting mixture was stirred for overnight at room temperature. The solution was quenched with aq. NH_4Cl solution and distilled water. Extracted with ethyl acetate and dried over $MgSO_4$. Filtered it and concentrated *in vacuo*. The residue was purified by flash column chromatography on silica gel.

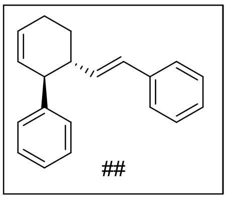

was prepared by General procedure D, Yield = 86%;

¹H NMR (400 MHz, $CDCl_3$) δ 7.32 – 7.25 (m, 6H), 7.22 – 7.15 (m, 4H), 6.17 (d, *J* = 1.7 Hz, 1H), 6.16 (s, 1H), 5.92 (dq, *J* = 9.8, 3.2 Hz, 1H), 5.69 (dq, *J* = 10.0, 2.4 Hz, 1H), 3.26 (dp, *J* = 8.4, 2.7 Hz, 1H), 2.43 (dddt, *J* = 10.9, 5.5, 4.3, 2.9 Hz, 1H), 2.23 (dtt, *J* = 6.6, 3.1, 2.1 Hz, 2H), 1.94 (dq, *J* = 12.4, 4.2 Hz, 1H), 1.69 (dddd, *J* = 13.0, 10.7, 9.2, 6.4 Hz, 1H).

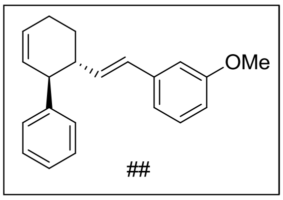

was prepared by General procedure D, Yield = 97%;

¹H NMR (400 MHz, $CDCl_3$) δ 7.32 – 7.23 (m, 2H), 7.23 – 7.13 (m, 4H), 6.86 (dt, *J* = 7.7, 1.2 Hz, 1H), 6.80 (dd, *J* = 2.6, 1.6 Hz, 1H), 6.73 (ddd, *J* = 8.2, 2.6, 0.9 Hz, 1H), 6.16 (d, *J* = 6.3 Hz, 1H), 6.14 (s, 1H), 5.91 (dq, *J* = 9.8, 3.1 Hz, 1H), 5.69 (dq, *J* = 10.0, 2.3 Hz, 1H), 3.79 (s, 3H), 3.26 (dp, *J* = 8.3, 2.6 Hz, 1H), 2.42 (dddd, *J* = 11.5, 9.1, 6.3, 3.0 Hz, 1H), 2.32 – 2.14 (m, 2H), 1.93 (dq, *J* = 12.4, 4.3 Hz, 1H), 1.69 (dddd, *J* = 13.0, 10.7, 9.1, 6.4 Hz, 1H).

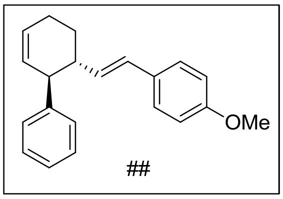

was prepared by General procedure D, Yield = 66%;

¹H NMR (400 MHz, $CDCl_3$) δ 7.29 – 7.24 (m, 2H), 7.20 – 7.15 (m, 5H), 6.79 (d, *J* = 8.8 Hz, 2H), 6.10 (d, *J* = 16.0 Hz, 1H), 6.01 (dd, *J* = 15.9, 7.3 Hz, 1H), 5.90 (dq, *J* = 9.6, 3.0, 2.6 Hz, 1H), 5.67 (dq, *J* = 10.0, 2.2 Hz, 1H), 3.77 (s, 3H), 3.23 (dp, *J* = 8.3, 2.7 Hz, 1H), 2.43 – 2.34 (m, 1H), 2.27 – 2.14 (m, 2H), 1.91 (dq, *J* = 12.5, 4.3 Hz, 1H), 1.66 (dddd, *J* = 13.0, 10.7, 9.1, 6.5 Hz, 1H).

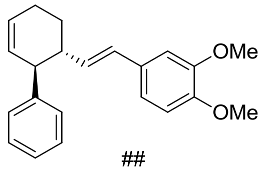

##

was prepared by General procedure D, Yield = 59%;

^{1}H NMR (400 MHz, $CDCl_3$) δ 7.27 – 7.23 (m, 2H), 7.21 – 7.16 (m, 3H), 6.79 (t, *J* = 1.2 Hz, 1H), 6.76 (d, *J* = 1.8 Hz, 1H), 6.75 (s, 1H), 6.08 (d, *J* = 16.0 Hz, 1H), 6.00 (dd, *J* = 15.9, 7.1 Hz, 1H), 5.89 (dq, *J* = 9.8, 3.3, 2.9 Hz, 1H), 5.67 (dq, *J* = 10.0, 2.2 Hz, 1H), 3.85 (s, 3H), 3.84 (s, 3H), 3.23 (dp, *J* = 8.0, 2.6 Hz, 1H), 2.43 – 2.33 (m, 1H), 2.25 – 2.17 (m, 2H), 1.91 (dq, *J* = 12.4, 4.3 Hz, 1H), 1.66 (dddd, *J* = 13.0, 10.5, 9.0, 6.6 Hz, 1H).

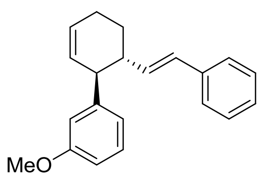

##

was prepared by General procedure D, Yield = 86%;

^{1}H NMR (400 MHz, $CDCl_3$) δ 7.26 – 7.24 (m, 4H), 7.20 – 7.14 (m, 2H), 6.80 – 6.77 (m, 1H), 6.75 – 6.71 (m, 2H), 6.18 (s, 1H), 6.17 (d, *J* = 3.3 Hz, 1H), 5.89 (dq, *J* = 9.7, 3.1, 2.6 Hz, 1H), 5.67 (dq, *J* = 10.0, 2.3 Hz, 1H), 3.76 (s, 3H), 3.23 (dp, *J* = 8.3, 2.7 Hz, 1H), 2.47 – 2.38 (m, 1H), 2.28 – 2.15 (m, 2H), 1.96 – 1.88 (m, 1H), 1.67 (dddd, *J* = 13.0, 10.7, 9.1, 6.4 Hz, 1H).

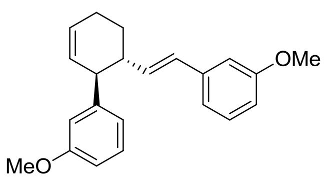

##

was prepared by General procedure D, Yield = 67%;

^{1}H NMR (400 MHz, $CDCl_3$) δ 7.17 (td, *J* = 7.5, 1.2 Hz, 1H), 7.16 (t, *J* = 7.9 Hz, 1H), 6.85 (d, *J* = 7.7 Hz, 1H), 6.79 – 6.75 (m, 2H), 6.74 – 6.69 (m, 3H), 6.15 (d, *J* = 2.0 Hz, 1H), 6.14 (s, 1H), 5.88 (dq, *J* = 9.7, 3.2 Hz, 1H), 5.65 (dq, *J* = 10.0, 2.3 Hz, 1H), 3.77 (s, 3H), 3.75 (s, 3H), 3.21 (dp, *J* = 8.4, 2.7 Hz, 1H), 2.45 – 2.36 (m, 1H), 2.28 – 2.12 (m, 2H), 1.91 (dq, *J* = 12.5, 3.8 Hz, 1H), 1.65 (dddd, *J* = 13.1, 10.6, 9.1, 6.5 Hz, 1H).

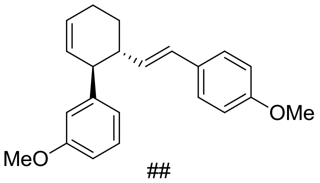

##

was prepared by General procedure D, Yield = 5.4%;

^{1}H NMR (400 MHz, $CDCl_3$) δ 7.21 – 7.13 (m, 3H), 6.81 – 6.68 (m, 5H), 6.11 (d, *J* = 16.0 Hz, 1H), 6.00 (dd, *J* = 15.9, 7.4 Hz, 1H), 5.87 (dq, *J* = 10.0, 3.5 Hz, 1H), 5.64 (dq, *J* = 10.0, 2.2 Hz, 1H), 3.76 (s, 3H), 3.75 (s, 3H), 3.19 (dt, *J* = 8.3, 2.7 Hz, 1H), 2.38 (dtd, *J* = 10.8, 7.8, 2.9 Hz, 1H), 2.22 – 2.15 (m, 2H), 1.89 (dq, *J* = 12.4, 4.2 Hz, 1H), 1.63 (dddd, *J* = 13.0, 10.6, 9.0, 6.6 Hz, 1H).

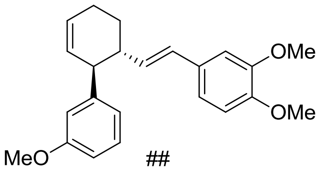

##

was prepared by modified the General Procedure D using NaH in DMF instead of nBuLi in THF, Yield = 26%;

^{1}H NMR (400 MHz, $CDCl_3$) δ 7.17 (td, *J* = 7.6, 0.7 Hz, 1H), 6.81 – 6.72 (m, 6H), 6.10 (d, *J* = 16.0 Hz, 1H), 6.01 (dd, *J* = 15.9, 7.4 Hz, 1H), 5.88 (dq, *J* = 10.1, 3.5 Hz, 1H), 5.65 (ddt, *J* = 10.0, 4.4, 2.2 Hz, 1H), 3.85 (s, 3H), 3.84 (s, 3H), 3.75 (s, 3H), 3.21 (dt, *J* = 8.5, 2.7 Hz, 1H), 2.39 (dtd, *J* = 10.3, 8.0, 2.9 Hz, 1H), 2.23 – 2.16 (m, 2H), 1.94 – 1.86 (m, 1H), 1.70 – 1.60 (m, 1H).

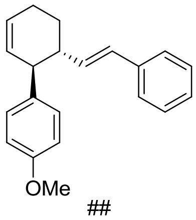

##

was prepared by General procedure D, Yield = 98%;

^{1}H NMR (400 MHz, $CDCl_3$) δ 7.27 – 7.22 (m, 4H), 7.19 – 7.13 (m, 1H), 7.09 (d, *J* = 8.5 Hz, 2H), 6.81 (d, *J* = 8.8 Hz, 2H), 6.16 (s, 1H), 6.15 (d, *J* = 1.1 Hz, 1H), 5.87 (dq, *J* = 9.7, 3.1 Hz, 1H), 5.65 (dq, *J* = 10.0, 2.3 Hz, 1H), 3.77 (s, 3H), 3.19 (dp, *J* = 8.4, 2.7 Hz, 1H), 2.36 (ddd, *J* = 14.4, 7.6, 3.4 Hz, 1H), 2.20 (dddd, *J* = 10.4, 4.8, 4.0, 2.0 Hz, 2H), 1.91 (dq, *J* = 12.4, 4.3 Hz, 1H), 1.66 (dddd, *J* = 13.0, 10.7, 9.1, 6.5 Hz, 1H).

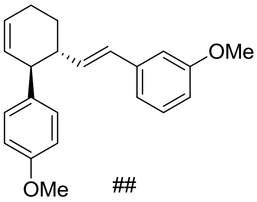

##

was prepared by General procedure D, Yield = 44%;

^{1}H NMR (400 MHz, $CDCl_3$) δ 7.16 (t, *J* = 7.9 Hz, 1H), 7.08 (d, *J* = 8.6 Hz, 2H), 6.85 (d, *J* = 7.7 Hz, 1H), 6.83 – 6.75 (m, 3H), 6.71 (ddd, *J* = 8.2, 2.6, 0.9 Hz, 1H), 6.13 (m, 2H), 5.87 (dq, *J* = 9.8, 3.2 Hz, 1H), 5.64 (dq, *J* = 10.0, 2.3 Hz, 1H), 3.78 (s, 3H), 3.76 (s, 3H), 3.18 (dt, *J* = 8.4, 2.7 Hz, 1H), 2.41 – 2.29 (m, 1H), 2.19 (tq, *J* = 6.9, 2.5 Hz, 2H), 1.90 (dq, *J* = 12.3, 3.9 Hz, 1H), 1.65 (dddd, *J* = 12.9, 10.7, 9.1, 6.5 Hz, 1H).

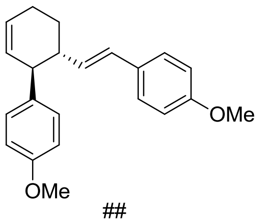

##

was prepared by General procedure D, Yield = 9%;

^{1}H NMR (400 MHz, $CDCl_3$) δ 7.17 (d, *J* = 8.7 Hz, 2H), 7.07 (d, *J* = 8.7 Hz, 2H), 6.79 (d, *J* = 8.8 Hz, 2H), 6.78 (d, *J* = 8.8 Hz, 2H), 6.09 (d, *J* = 15.9 Hz, 1H), 5.99 (dd, *J* = 16.0, 7.4 Hz, 1H), 5.85 (dq, *J* = 10.1, 3.5 Hz, 1H),

5.63 (dq, J = 10.0, 2.1 Hz, 1H), 3.76 (s, 3H), 3.76 (s, 3H), 3.16 (dt, J = 8.3, 2.7 Hz, 1H), 2.37 – 2.28 (m, 1H), 2.22 – 2.14 (m, 2H), 1.93 – 1.83 (m, 1H), 1.63 (ddd, J = 12.9, 7.2, 3.1 Hz, 1H).

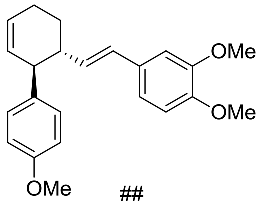

##

was prepared by modified the General Procedure D using NaH in DMF instead of nBuLi in THF, Yield = 46%;

^{1}H NMR (400 MHz, $CDCl_3$) δ 7.08 (d, J = 8.7 Hz, 2H), 6.82 – 6.75 (m, 5H), 6.09 (d, J = 15.9 Hz, 1H), 6.00 (dd, J = 15.9, 7.3 Hz, 1H), 5.87 (dq, J = 9.9, 3.3 Hz, 1H), 5.64 (ddt, J = 10.1, 3.9, 2.2 Hz, 1H), 3.86 (s, 3H), 3.84 (s, 3H), 3.76 (s, 3H), 3.18 (dt, J = 8.5, 2.7 Hz, 1H), 2.33 (dtd, J = 10.5, 8.1, 2.9 Hz, 1H), 2.22 – 2.15 (m, 2H), 1.89 (dq, J = 12.4, 4.2 Hz, 1H), 1.64 (dddd, J = 13.0, 10.3, 8.9, 6.6 Hz, 1H).

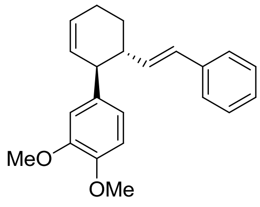

##

was prepared by General procedure D, Yield = 36%;

^{1}H NMR (400 MHz, $CDCl_3$) δ 7.25 – 7.23 (m, 4H), 7.18 – 7.12 (m, 1H), 6.76 (d, J = 8.1 Hz, 1H), 6.72 (d, J = 2.0 Hz, 1H), 6.68 (d, J = 1.9 Hz, 1H), 6.15 (d, J = 1.0 Hz, 1H), 6.14 (s, 1H), 5.88 (ddd, J = 9.6, 6.0, 2.6 Hz, 1H), 5.66 (dq, J = 10.0, 2.3 Hz, 1H), 3.83 (s, 3H), 3.80 (s, 3H), 3.17 (dp, J = 8.5, 2.7 Hz, 1H), 2.42 – 2.30 (m, 1H), 2.20 (ddt, J = 8.6, 4.4, 2.6 Hz, 2H), 1.95 – 1.87 (m, 1H), 1.66 (dddd, J = 13.0, 10.8, 9.2, 6.4 Hz, 1H).

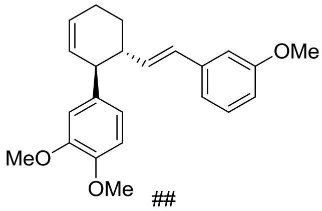

##

was prepared by General procedure D, Yield = 70%;

^{1}H NMR (400 MHz, $CDCl_3$) δ 7.15 (t, J = 7.9 Hz, 1H), 6.84 (dt, J = 7.6, 1.1 Hz, 1H), 6.78 (dd, J = 2.6, 1.6 Hz, 1H), 6.76 (d, J = 8.1 Hz, 1H), 6.73 – 6.67 (m, 3H), 6.15 – 6.11 (m, 2H), 5.88 (dq, J = 9.7, 3.2 Hz, 1H), 5.66 (dq, J = 10.0, 2.3 Hz, 1H), 3.83 (s, 3H), 3.80 (s, 3H), 3.77 (s, 3H), 3.17 (dp, J = 8.3, 2.7 Hz, 1H), 2.35 (dddd, J = 11.3, 9.0, 6.1, 3.0 Hz, 1H), 2.20 (ddt, J = 6.5, 4.4, 2.6 Hz, 2H), 1.91 (dq, J = 12.4, 3.9 Hz, 1H), 1.65 (dddd, J = 13.0, 10.7, 9.2, 6.4 Hz, 1H).

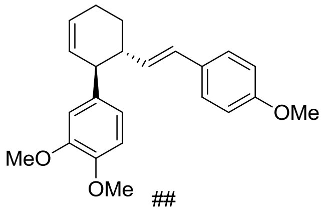

##

was prepared by General procedure D, Yield = 10%;

^{1}H NMR (400 MHz, $CDCl_3$) δ 7.17 (d, *J* = 8.6 Hz, 2H), 6.78 (d, *J* = 8.8 Hz, 2H), 6.75 (s, 1H), 6.71 (d, *J* = 1.9 Hz, 1H), 6.67 (d, *J* = 1.9 Hz, 1H), 6.08 (d, *J* = 15.7 Hz, 1H), 5.99 (dd, *J* = 16.0, 7.4 Hz, 1H), 5.92 – 5.82 (m, 1H), 5.65 (dq, *J* = 9.6, 2.3 Hz, 1H), 3.83 (s, 3H), 3.80 (s, 3H), 3.76 (s, 3H), 3.20 – 3.10 (m, 1H), 2.39 – 2.26 (m, 1H), 2.19 (dt, *J* = 6.0, 2.9 Hz, 2H), 1.95 – 1.84 (m, 1H), 1.70 – 1.57 (m, 1H).

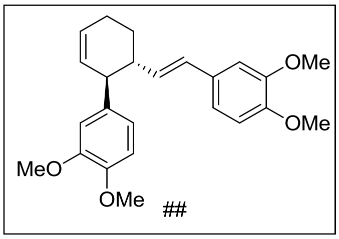

##

was prepared by General procedure D, Yield = 34%;

^{1}H NMR (599 MHz, $CDCl_3$) δ 6.81 – 6.75 (m, 2H), 6.78 – 6.72 (m, 2H), 6.71 (dd, *J* = 8.2, 1.8 Hz, 1H), 6.68 (d, *J* = 1.8 Hz, 1H), 6.07 (d, *J* = 15.9 Hz, 1H), 6.00 (dd, *J* = 15.9, 7.4 Hz, 1H), 5.88 (ddt, *J* = 9.6, 4.9, 2.8 Hz, 1H), 5.66 (dd, *J* = 10.0, 2.1 Hz, 1H), 3.85 (s, 3H), 3.84 (s, 3H), 3.83 (s, 3H), 3.80 (s, 3H), 3.16 (dt, *J* = 8.2, 2.6 Hz, 1H), 2.33 (dtd, *J* = 10.7, 7.7, 2.8 Hz, 1H), 2.20 (ddp, *J* = 6.5, 4.5, 2.4 Hz, 2H), 1.90 (dq, *J* = 12.3, 4.5 Hz, 1H), 1.65 (dtd, *J* = 12.9, 10.1, 6.2 Hz, 1H).

Experimental procedure for Supplementary Figure 10B

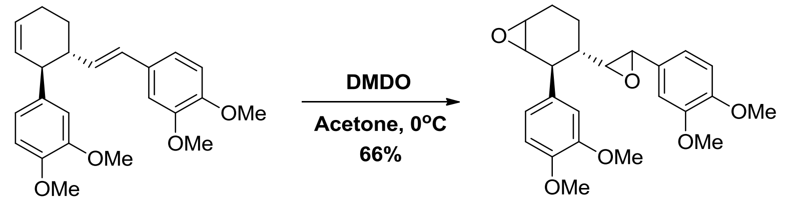

##

To a round bottom flask containing NMac1(1.0 eq.) was added the prepared solution of DMDO(0.044 M in acetone, 7.0 eq.) at 0 ^{o}C under Ar. The resulting mixture was stirred for 1h, and then solvent was removed *in vacuo*. The residue was purified by flash column chromatography on silica gel to obtain ## in 66% yield with four diastereomers as inseparable mixture.

^{1}H NMR (400 MHz, Chloroform-*d*) δ 6.94 (dd, *J* = 3.7, 2.0 Hz, 1H), 6.91 (s, 1H), 6.89 (s, 1H), 6.85 (d, *J* = 2.5 Hz, 1H), 6.84 – 6.80 (m, 4H), 6.80 – 6.78 (m, 2H), 6.76 (s, 1H), 6.75 – 6.72 (m, 3H), 6.66 – 6.56 (m, 4H), 6.40 (d, *J* = 2.0 Hz, 1H), 6.39 – 6.36 (m, 1H), 6.34 (d, *J* = 2.0 Hz, 2H), 6.32 (dd, *J* = 8.3, 1.9 Hz, 1H), 6.27 (d, *J* = 2.0 Hz, 1H), 3.95 – 3.82 (m, 48H), 3.68 (d, *J* = 9.0 Hz, 8H), 3.59 (dd, *J* = 12.6, 2.2 Hz, 2H), 3.37 (s, 3H), 3.29 (dd, *J* = 8.3, 3.3 Hz, 3H), 3.16 (dd, *J* = 17.0, 3.8 Hz, 3H), 2.90 – 2.83 (m, 4H), 2.81 – 2.78 (m, 3H), 2.56 (ddd, *J* = 14.2, 8.5, 2.1 Hz, 3H), 2.39 – 2.31 (m, 3H), 2.00 – 1.88 (m, 5H), 1.86 (d, *J* = 2.4 Hz, 3H).

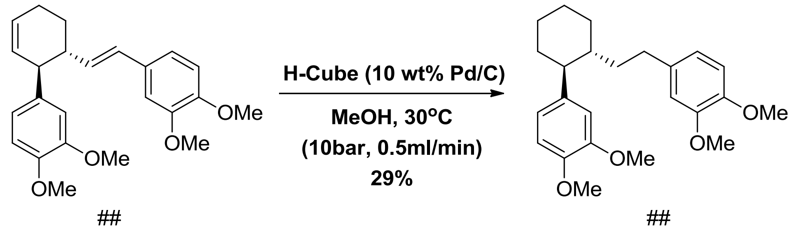

##

The solution of NMac1(7.71 mg, 0.0203 mmol) in MeOH(2 mL) underwent hydrogenolysis with a shaker-type Parr Hydrogenator under H_2(10 bar), 10wt% Pd/C(flowing rate 0.5 mL/min, 30~33 °C). The reaction mixture was evaporated *in vacuo* and purified by flash column chromatography on silica gel to obtain ## in 29% yield.

^{1}H NMR (599 MHz, $CDCl_3$) δ 6.75 (d, *J* = 8.1 Hz, 1H), 6.69 (d, *J* = 8.1 Hz, 1H), 6.61 (d, *J* = 8.1 Hz, 1H), 6.59 (s, 1H), 6.50 (d, *J* = 8.1 Hz, 1H), 6.45 (s, 1H), 3.83 (s, 3H), 3.81 (s, 3H), 3.80 (s, 3H), 3.76 (s, 3H), 2.53 (ddd, *J* = 14.0, 9.6, 4.6 Hz, 1H), 2.29 (dt, *J* = 13.8, 8.6 Hz, 1H), 2.14 (dt, *J* = 11.2, 5.6 Hz, 1H), 2.03 (d, *J* = 13.2 Hz, 1H), 1.84 – 1.73 (m, 3H), 1.45 (ddtd, *J* = 29.7, 14.8, 7.3, 4.1 Hz, 2H), 1.38 – 1.35 (m, 1H), 1.35 – 1.29 (m, 2H), 1.17 (dtd, *J* = 13.5, 9.0, 4.7 Hz, 1H), 1.07 (qd, *J* = 12.7, 3.4 Hz, 1H).

Experimental procedure for Supplementary Figure 10C

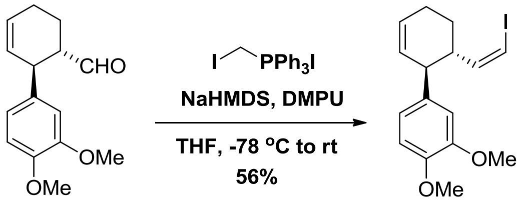

##

To a stirred solution of (Iodomethyl)triphenylphosphonium iodide(1.5 eq.) in THF was added NaHMDS(1.0M in THF, 1.5 eq.) at 0 °C under Ar. Then the resulting mixture was allowed to warm up to room temperature and stirred for 30min. Then solution was cooled down to -78 °C again and the solution *cis*-aldehyde(1.0 eq.) in THF/DMPU=3/1 was added by cannula under Ar. The resulting mixture was stirred for overnight. The solution was quenched with aq. NH_4Cl solution and distilled water. Extracted with ethyl acetate and dried over $MgSO_4$. Filtered it and concentrated *in vacuo*. The residue was purified by flash column chromatography on silica gel to obtain ## in 56% yield as (*Z*)-isomer..

^{1}H NMR (600 MHz, $CDCl_3$) δ 6.76 (d, *J* = 8.2 Hz, 1H), 6.73 (d, *J* = 1.8 Hz, 1H), 6.70 (dd, *J* = 8.2, 1.9 Hz, 1H), 6.10 (s, 1H), 6.09 (d, *J* = 2.9 Hz, 1H), 5.85 (ddt, *J* = 9.6, 4.7, 2.5 Hz, 1H), 5.63 (dd, *J* = 10.0, 2.0 Hz, 1H), 3.87 (s, 3H), 3.84 (s, 3H), 3.19 (dt, *J* = 7.9, 2.6 Hz, 1H), 2.72 – 2.63 (m, 1H), 2.24 (dddt, *J* = 18.3, 9.2, 6.1, 3.2 Hz, 1H), 2.13 (dddd, *J* = 16.2, 8.0, 4.2, 2.0 Hz, 1H), 1.77 (ddt, *J* = 12.9, 5.9, 4.0 Hz, 1H), 1.60 – 1.50 (m, 1H).

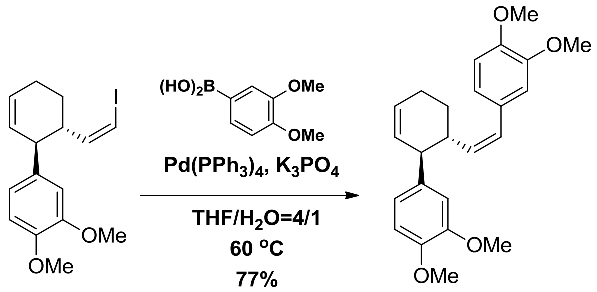

##

To a stirred solution of vinyl iodide(1.0 eq.) in THF/water=4/1 was added 3,4-dimethoxyphenylboronic acid(2.0 eq.), K_3PO_4(2.0 eq.) and $Pd(PPh_3)_4$(0.05 eq.) at room temperature under Ar. Then the resulting mixture was stirred for 1h at 60 °C. The solution was diluted with ethyl acetate and washed with aq. NH_4Cl solution and distilled water. Extracted with ethyl acetate and dried over $MgSO_4$. Filtered it and concentrated *in vacuo*. The residue was purified by flash column chromatography on silica gel to obtain ## in 77% yield as (*Z*)-isomer.

^{1}H NMR (400 MHz, $CDCl_3$) δ 6.70 (d, *J* = 8.2 Hz, 1H), 6.68 (d, *J* = 8.3 Hz, 1H), 6.63 (dd, *J* = 8.2, 2.0 Hz, 1H), 6.54 (d, *J* = 2.0 Hz, 1H), 6.40 (s, 1H), 6.39(dd, *J* = 8.5, 2.2 Hz, 1H), 6.25 (d, *J* = 11.3 Hz, 1H), 5.82 (dq, *J* = 9.9, 3.4 Hz, 1H), 5.63 (dq, *J* = 10.1, 2.2 Hz, 1H), 5.49 (dd, *J* = 11.5, 10.3 Hz, 1H), 3.82 (s, 3H), 3.81 (s, 3H), 3.74 (s, 3H), 3.71 (s, 3H), 3.13 (dt, *J* = 8.6, 2.7 Hz, 1H), 2.88 – 2.75 (m, 1H), 2.18 – 2.11 (m, 2H), 1.85 – 1.77 (m, 1H), 1.62 (dddd, *J* = 13.2, 11.0, 9.2, 6.9 Hz, 1H).

Experimental procedure for Supplementary Figure 10D

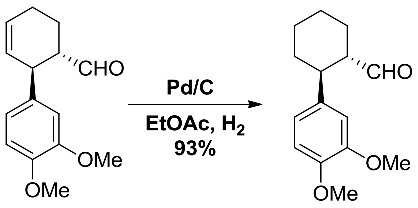

##

To a stirred solution of cyclohexene(1.0 eq.) in ethyl acetate was added 10 wt% Pd/C(0.1eq.) at room temperature under Ar. The Ar atmosphere was substituted with H_2 atmosphere. The resulting mixture was stirred for 6h. The resulting mixture was filtered through silica/Celite pad and washed with ethyl acetate a few times. The filtrate was concentrated *in vacuo* and the residue was purified by flash column chromatography on silica gel to obtain ## in 93% yield.

^{1}H NMR (599 MHz, Chloroform-*d*) δ 9.39 (d, *J* = 3.1 Hz, 1H), 6.81 – 6.75 (m, 1H), 6.74 – 6.66 (m, 2H), 3.84 (s, 3H), 3.82 (s, 3H), 2.66 (td, *J* = 11.7, 3.5 Hz, 1H), 2.56 – 2.47 (m, 1H), 1.94 – 1.79 (m, 4H), 1.51 – 1.41 (m, 1H), 1.41 – 1.31 (m, 3H).

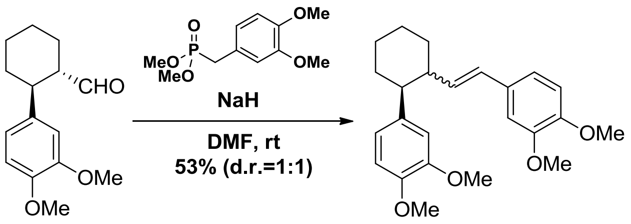

##

To a stirred solution of dimethyl (3,4-dimethoxybenzyl)phosphonate(2.0 eq.) in DMF was added NaH(60% in mineral oil, 1.95 eq.) at 0 °C under Ar. The resulting mixture was stirred for 30min at room temperature. The solution of *cis*-aldehyde(1.0 eq.) in DMF was added to the prepared ylide solution by cannula under Ar. The resulting mixture was stirred for overnight at room temperature. The solution was quenched with aq. NH_4Cl solution and distilled water. Extracted with ethyl acetate and dried over $MgSO_4$. Filtered it and concentrated *in vacuo*. The residue was purified by flash column chromatography on silica gel to obtain ## in 53% yield with two inseparable diastereomers.

^{1}H NMR (400 MHz, Chloroform-*d*) δ 7.07 – 6.98 (m, 4H), 6.91 (s, 2H), 6.84 (d, *J* = 8.2 Hz, 2H), 6.74 (d, *J* = 8.0 Hz, 1H), 6.72 – 6.65 (m, 5H), 6.04 (d, *J* = 15.9 Hz, 1H), 5.77 (dd, *J* = 15.9, 6.9 Hz, 1H), 3.93 (s, 6H), 3.88 (s, 6H), 3.82 (s, 3H), 3.81 (s, 9H), 2.35 – 2.21 (m, 2H), 1.87 (td, *J* = 19.9, 16.5, 9.8 Hz, 5H), 1.38 (t, *J* = 10.8 Hz, 9H).

Experimental procedure for Supplementary Figure 10E

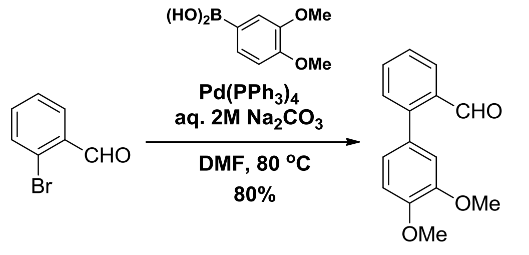

##

To a stirred solution *o*-bromobenzaldehyde(1.0 eq.) in DMF was added 3,4-dimethoxyphenylboronic acid(2.0

eq.), aq. 2M Na_2CO_3(3.0 eq.) and $Pd(PPh_3)_4$(0.01 eq.) at room temperature under Ar. Then the resulting mixture was stirred for overnight at 80 °C. The solution was diluted with ethyl acetate and washed with aq. NH_4Cl solution and distilled water. Extracted with ethyl acetate and dried over $MgSO_4$. Filtered it and concentrated *in vacuo*. The residue was purified by flash column chromatography on silica gel to obtain ## in 80% yield.

^{1}H NMR (300 MHz, $CDCl_3$) δ 9.98 (s, 1H), 7.98 (d, *J* = 7.9 Hz, 1H), 7.61 (td, *J* = 7.5, 1.4 Hz, 1H), 7.45 (t, *J* = 8.0 Hz, 2H), 6.98 – 6.85 (m, 3H), 3.93 (s, 3H), 3.89 (s, 3H).

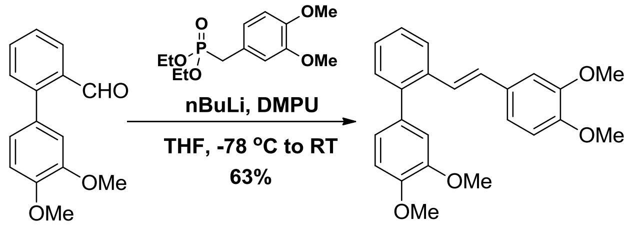

##

Prepared from ## using General Procedure D in 63% yield.

^{1}H NMR (599 MHz, $CDCl_3$) δ 7.70 (d, *J* = 7.6 Hz, 1H), 7.33 (d, *J* = 7.1 Hz, 2H), 7.30 (d, *J* = 6.2 Hz, 1H), 7.03 – 6.91 (m, 6H), 6.89 (d, *J* = 1.6 Hz, 1H), 6.80 (d, *J* = 8.3 Hz, 1H), 3.92 (s, 3H), 3.86 (s, 3H), 3.84 (s, 3H), 3.83 (s, 3H).

Alternative procedure for NMac1 in large scale synthesis

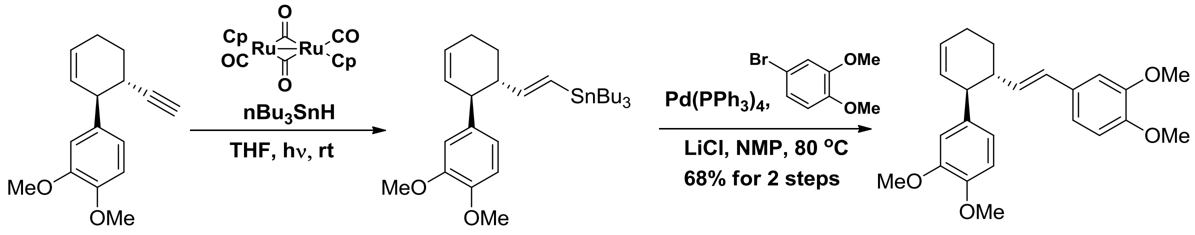

##

To a stirred solution alkyne[1](476 mg, 1.96 mmol, 1.0 eq.) in THF(10 mL) was added Ru catalyst(87 mg, 0.196 mmol, 0.1 eq.) at room temperature under Ar. Then the resulting solution was degassed for 15min and tributyltin hydride(1.09 mL, 3.93 mmol, 2.0 eq.) was added. The mixture was stirred for 2h under the irradiation of white LED light. The solvent was removed by rotary evaporator. Filtered through short silica pad and concentrated *in vacuo*. The crude product was used for next step without further purification.

To a stirred solution vinyl stannane(894 mg, 1.67 mmol, 1.0 eq.) in NMP(8 mL) was added 4-bromo-1,2-dimethoxybenzene(0.295 mL, 2.01 mmol, 1.2 eq.), LiCl (85 mg, 2.01 mmol, 1.2 eq.) and $Pd(PPh_3)_4$ (391mg, 0.335 mmol, 0.2 eq.) at room temperature under Ar. Then the resulting mixture was stirred for 5h at 80 °C. The solution was diluted with ethyl acetate and washed with brine and distilled water. Extracted with ethyl acetate and dried over $MgSO_4$. Filtered it and concentrated *in vacuo*. The residue was purified by flash column chromatography on silica gel to obtain ## in 80% yield.

^{1}H NMR (599 MHz, $CDCl_3$) δ 6.81 – 6.75 (m, 2H), 6.78 – 6.72 (m, 2H), 6.71 (dd, *J* = 8.2, 1.8 Hz, 1H), 6.68 (d, *J* = 1.8 Hz, 1H), 6.07 (d, *J* = 15.9 Hz, 1H), 6.00 (dd, *J* = 15.9, 7.4 Hz, 1H), 5.88 (ddt, *J* = 9.6, 4.9, 2.8 Hz, 1H), 5.66 (dd, *J* = 10.0, 2.1 Hz, 1H), 3.85 (s, 3H), 3.84 (s, 3H), 3.83 (s, 3H), 3.80 (s, 3H), 3.16 (dt, *J* = 8.2, 2.6 Hz, 1H), 2.33 (dtd, *J* = 10.7, 7.7, 2.8 Hz, 1H), 2.20 (ddp, *J* = 6.5, 4.5, 2.4 Hz, 2H), 1.90 (dq, *J* = 12.3, 4.5 Hz, 1H), 1.65 (dtd, *J* = 12.9, 10.1, 6.2 Hz, 1H).

[1] Corresponding alkyne was prepared from aldehyde from following procedure.
J. Chu, D. H. Suh, G. Lee, A. -R. Han, S. W. Chae, H. J. Lee, E. -K. Seo, H. -J. Lim, *J. Nat. Prod.* **2011**, *74*, 1817.
